# c-Src Increases the Sensitivity to TKIs in the EGFR-Mutant Lung Adenocarcinoma

**DOI:** 10.3389/fonc.2021.602900

**Published:** 2021-07-22

**Authors:** Weili Min, Chenyang He, Shuqun Zhang, Yang Zhao

**Affiliations:** Department of Oncology, The Second Affiliated Hospital of Xi’an Jiaotong University, Xi’an, China

**Keywords:** c-Src, caspase-8, non-small cell lung cancer, tyrosine kinase inhibitor, resistance

## Abstract

c-Src and the epidermal growth factor receptor (EGFR) are key apical kinases that govern cell responses to microenvironmental cues. How c-Src affects EGFR-related signaling and targeted therapy remains elusive. Initially, caspase-8 phosphorylated at tyrosine 380 by c-Src predominantly enhancing c-Src activation to facilitate metastasis through attaining epithelial-mesenchymal transition (EMT) phenotype in lung adenocarcinoma. Mechanistically, the linkage of c-Src SH2 domain with phosphotyrosine 380 of caspase-8 and SH3 domain with “PDEP” motif of caspase-8 overactivates c-Src as compared with other c-Src-partner proteins. c-Src is incapable of triggering EGFR-related signaling. This is reflected by the levels of phosphotyrosine 1068, 1086, and 1145, which have no impact on c-Src activation. Tyrosine kinase inhibitors (TKIs) suppress EGFR-related signaling to yield cell deaths of lung adenocarcinoma by both necroptosis and intrinsic apoptosis. Given that c-Src activation is frequent in lung adenocarcinoma, blocking c-Src activation through dasatinib can seal the survival-signaling-related phosphotyrosines of EGFR by its SH2 domain, which in turn increases the antitumor activity of TKIs in EGFR-mutant lung adenocarcinoma. Collectively, c-Src inactivation by dasatinib administration sensitizes EGFR-mutant lung adenocarcinoma to TKIs.

## Highlights

- c-Src exclusively phosphorylated caspase-8 at tyrosine 380. Caspase-8 was predominant for c-Src overactivation by phosphotyrosine 380 and “PDEP” motif docking to SH2 domain and SH3 domain of c-Src in lung adenocarcinoma;
- c-Src inactivation through dasatinib was able to seal the survival-signaling-related phosphotyrosines of EGFR to increase the TKIs-induced necroptosis in the EGFR-mutant lung adenocarcinoma.

## Introduction

Non-small cell lung cancer (NSCLC) is the leading cause of cancer death worldwide, with a surprising increase in the incidence of lung adenocarcinoma; traditional chemotherapeutic drugs are only modestly effective (1–4). Recent advances with targeted therapies have provided a marked benefit to subsets of patients whose tumors harbor specific genetic abnormalities (5, 6). In particular, lung adenocarcinomas with mutations in the gene encoding the epidermal growth factor receptor (EGFR) are uniquely sensitive to EGFR blockade with specific tyrosine kinase inhibitors (TKIs) (7, 8). Majority of cancers with EGFR-sensitive mutations achieve the marked and durable responses to treatment with EGFR TKIs, including gefitinib or erlotinib. However, lung adenocarcinoma inevitably acquires resistance to these inhibitors after approximately 1 year. Multiple mechanisms of acquired resistance to first- and second-generation EGFR-TKIs have been identified thus far (7), in which 30% of EGFR TKI resistance was because of an unknown mechanism (8). This sheds light on the lack of research on the underlying mechanism of EGFR TKIs, which must be investigated to enhance the therapeutic potency in lung adenocarcinoma.

Caspase-8, an apical sensory protease, is recruited into the death-inducing signaling complexes following death-related receptor ligation to initiate an extrinsic apoptosis cascade (9, 10). The inactivating mutation of caspase-8 is surprisingly infrequent among various human cancers (11–13). The study that reported the association of caspase-8 to adhesion and metastasis of human tumors is actually recent (14–16). It is of note that caspase-8 has also been reported to be involved in the focal adhesion complexes (17). The interplay between caspase-8 and c-Src is confirmed by the observation that cells stimulated with the survival-promoting factors lead to c-Src–mediated caspase-8 phosphorylation on tyrosine 380 (pY380 caspase-8 or p-Casp8), which then inhibits its apoptotic function (18–20). Consistently, we found that p-Casp8 reversely activated c-Src (pY416 c-Src or p-Src) *via* docking of phosphotyrosine 380 to the SH2 domain to restrain chemotherapy efficacy in lung adenocarcinoma (21). c-Src overactivation was ubiquitously detected in human tumors and involved in the resistance of TKIs (7, 22). Therefore, it is of interest to explore the interaction between caspase-8 and c-Src and their effect on the clinical efficacy of TKIs in EGFR-mutant lung adenocarcinoma.

In our study, caspase-8 phosphorylated by c-Src predominantly enhanced c-Src activation to facilitate metastasis through attaining EMT phenotypic features in lung adenocarcinoma. We found that EGFR activation and c-Src activation did not mutually interact with one another. TKIs suppressed EGFR-related signaling to yield cell deaths of lung adenocarcinoma by necroptosis and intrinsic apoptosis. Surprisingly, c-Src inactivation through caspase-8 knockdown or dasatinib was able to block the survival-signaling-related tyrosine phosphorylation of EGFR, which, in turn, increased the antitumor activity of TKIs in EGFR-mutant lung adenocarcinoma.

## Materials and Methods

### Ethics Approval and Consent to Participate

The procedures of this study, which included seven references, were approved by the Ethics Committee of the Second Affiliated Hospital of Xi’an Jiaotong University. The experiments were performed upon receiving written consent from each subject. The study methodologies conformed to the standards set by the Declaration of Helsinki.

### Patients and Treatments

Human lung adenocarcinoma and adjacent paracancerous tissues (≥ 2.0 cm from the primary tumor site) from 84 patients were collected following surgeries at the Department of Pathology of the First and Second Affiliated Hospital of Xi’an Jiaotong University from 2009 to 2012. Lung adenocarcinoma was determined by two individual pathologists and classified as pathological stages I to IIIA according to the American Joint Committee on Cancer 2018 (AJCC 2018). Parallel to this, tissues from metastatic lung adenocarcinomas with EGFR-sensitive mutations of patients from our cancer center were retrospectively collected. EGFR mutation was performed by the Amplification-refractory mutation system (ARMS) in Big Science (China, HuaDa gene). The patients with EGFR-mutant lung adenocarcinoma received the first-generation TKIs gefitinib or erlotinib, in accordance with the guidelines. The response to treatment, including complete remission (CR), partial remission (PR), stable disease (SD) and progressive disease (PD), was evaluated according to response evaluation criteria in solid tumors (RECIST 1.1) until disease progression. The eligible patients were routinely scheduled for lifelong follow-up at the outpatient clinic every 3 months during the first 2 years and every 6 months for the next 3 years. Whenever recurrent or metastatic events were suspected, radiologic, endoscopic, and histologic confirmation was compulsory. The calculation of duration of response started at the date of treatment and ended at the date of the following events: recurrence, disease progression, or oncological death. The calculation of overall survival (OS) started at the date of treatment and ended at the date of death. Unless it was reported here, no participants were lost during follow-up. The study was approved by the Ethics Committee of the Second Affiliated Hospital of Xi’an Jiaotong University. Informed consent was obtained from the patients before the study implementation.

### Cell Culture, DNA/Short Hairpin RNA Transfection, and Stable Cell Line Generation

Lung adenocarcinoma cell lines, including A549 and National Cancer Institute (NCI)-H522, were kind gifts from Chen Huang from the Department of Cell Biology, Xi’an Jiaotong University, Shaanxi Province, P.R. China. H1650, H3255 and PC9 cells were purchased from the American Type Culture Collection (ATCC) and cultured in RPMI 1640 supplemented with 10% fetal bovine serum (FBS, Hyclone Laboratories Inc., Logan, UT, USA) and penicillin/streptomycin/L-glutamine (Sigma-Aldrich, St. Louis, MO, USA). The cell lines in our study were authenticated by short tandem repeat (STR) analysis. To observe the morphological features of EMT, we cultured cells for 5 days on fibronectin (10 μg/ml)-coated dishes. EGFR-nonaddictive lung adenocarcinoma cells were assayed for expression and viability following treatment with reagents or drugs at 24 h after attachment to the fibronectin-coated dishes. EGFR-addictive lung adenocarcinoma cells were not attached on fibronectin-coated dishes for 24 h. The knockdown of Fyn, Yes, Lyn, Hck, EGFR, Her-2, Her-3, Her-4, FAK, and caspase-8 or c-Src was performed using lentivirus-delivered short hairpin RNAs (shRNAs; GenePharma, Shanghai, China), with the shRNAs corresponding to the siRNAs. Following lentiviral transfection, stable cell lines were selected *via* culturing in the presence of 500 μg/ml G418 (Grand Island Biological Company, Waltham, MA, USA). The open reading frames of genes of interest, including Fgr, Lck, Blk, wild-type caspase-8, EGFR and its mutants, and c-Src and its mutants, were cloned into the MSCV-IRES-zeo plasmid with a hemagglutinin (HA) tag to allow the expression of HA-tagged proteins. The subsequent DNA was transfected into packaging cells. Virus-containing supernatants were removed, and debris was pelleted by centrifugation. Target cells were cultured in virus-containing supernatants for 48 h before selection for a stable cell line with 10 mg/ml zeocin (Invitrogen, Waltham, MA, USA) for 14 days.

## Results

### Tyrosine 380 of Caspase-8 Was Pivotal for Lung Adenocarcinoma Metastasis Through EMT

**Figure 1:**
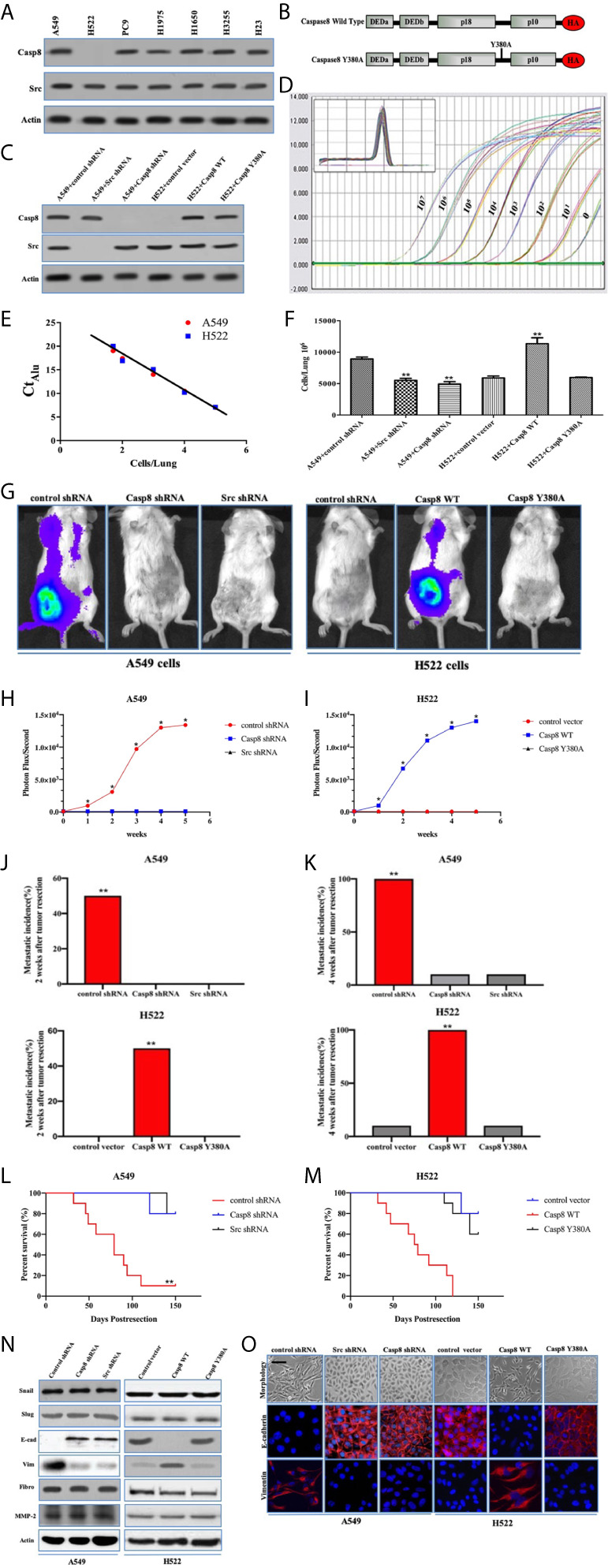
Tyrosine 380 of Caspase-8 was pivotal for lung adenocarcinoma metastasis through EMT. **(A)** Immunoblotting analysis of Caspase-8, c-Src and β-actin in various human lung adenocarcinoma cell lines, A549, NCI-H522, NCI-H1975, NCI-H1650, NCI-H3255, NCI-H23 and PC9. **(B)** Schematic representation of various mutants of Caspase-8 that were used in this study. **(C)** Immunoblotting analysis of Caspase-8, c-Src and β-actin in A549 + control/Src/Casp8 shRNA and H522 + control vector/Casp8 WT/Casp8 Y380A. **(D)** Alu PCR was used to amplify alu repeat sequences in human (A549 cells) DNA. Quantitative real-time alu PCR was performed on genomic DNA extracted from the indicated number of A549 cells serially diluted into individual chick embryo lung homogenates. **(E)** Real-time quantitative alu PCR was used to generate a standard curve from A549 cells/lung by plotting the Ct against the number of cells per lung. **(F)** A quantitative analysis of spontaneous metastasis in the chick embryo using different human tumor cells. *vs*. control, **p < 0.01. **(G–I)** Ectopically grown A549/H522 tumors were surgically removed. Biweekly quantification of bioluminescence showed accelerated tumor growth and increased spontaneous metastasis in the mice implanted with A549 + control shRNA **(H)** and H522 + Casp8 WT **(I)** (*p < 0.05). Data were presented as mean ± SD. **(J, K)** Total cumulative incidences of metastasis confirmed by immunostaining in the tumor-implanted mice cohorts by 2 or 4 weeks after tumor removal (**p < 0.01). Metastatic events were confirmed by immunostaining in various organs of BALB/c nude mice. Tissue sections were scored as positive or negative based on the presence or absence of detectable metastasis. **(L, M)** Kaplan-Meier survival curves for each group of mice with A549 cells **(L)** and H522 cells **(M)**. **(N)** Immunoblotting analysis of Snail, Slug, E-cadherin (E-cad), Vimentin (Vim), Fibronectin (Fibro), MMP-2 and β-actin in A549 + control/Src/Casp8 shRNA and H522 + control vector/Casp8 WT/Casp8 Y380A. **(O)** Immunofluorescence of E-cadherin and Vimentin. Decreased E-cadherin and increased Vimentin in the Casp8^+^Src^+^ cells with spindle and dendritic shapes (scale bars = 50 μm).

The recent evidence has been reported that supports the role of caspase-8 in tumor cell migration under the nonapoptotic condition (15, 17, 23, 24). Our team found that c-Src phosphorylated caspase-8 at tyrosine 380 hampered the apoptosis of caspase-8 responding to chemotherapy in lung adenocarcinoma (25). We initially probed the interactive effects of caspase-8 and c-Src on the aggressive properties of lung adenocarcinoma. The expressions of caspase-8 and c-Src were examined using immunoblotting in the lung adenocarcinoma-derived cell lines, including A549, H522, PC9, H1975, H1650, H3255, and H23 (**Figure 1A**). Casp8^−^Src^+^ H522 and Casp8^+^Src^+^A549 with wild type (WT) EGFR were selected as experimental cell lines. Given the role of tyrosine 380 of caspase-8, we constructed the hemagglutinin (HA)-tagged wild type/Y380A mutant caspase-8 (**Figure 1B**) and reconstituted the physiological level of these constructions in the caspase-8-deficient H522 cells (**Figure 1C**). In addition, the lentivirus-delivered shRNAs of caspase-8 and c-Src were applied to knockdown endogenous caspase-8 and c-Src in A549 cells (**Figure 1C**).

The chick embryo model has been useful in investigating angiogenesis and tumorigenesis *in vivo* (26, 27). To approximate the actual number of tumor cells in each tissue sample, a standard curve was generated through quantitative real-time PCR amplification of genomic DNA extracted from a serial dilution of A549/H522 cells mixed with individual chick lung homogenates (**Figure 1D**). By interpolating the alu signal from real-time PCR with the standard curve (**Figure 1E**), the actual number of tumor cells/lung could be determined over a linear range. Knockdown of endogenous caspase-8 or c-Src by lentivirus-delivered shRNAs of caspase-8 and c-Src efficiently decreased A549 cells metastasis as compared with control shRNA (**Figure 1F**). A disproportionate increase in the metastasis was also detected in the H522 cells re-expressing WT caspase-8 (Casp8 WT) (**Figure 1F**), whereas Y380A mutation in the holoprotein of caspase-8 obviously attenuated the metastasis of H522 cells (**Figure 1F**). We subsequently explored the role of caspase-8 and c-Src in spontaneous distant metastasis after primary tumors that weighed the same size were removed from nude mice. As measured by bioluminescence (**Figure 1G**), mice implanted with A549 + control shRNA cells and H522 + Casp8 WT cells had a significantly increased tumor burden, and metastasis occurred in all 10 mice in the group (**Figures 1H, I**). Markedly increased metastatic incidences and extended tumor distributions were observed in the mice inoculated with A549 + control shRNA cells and H522 + Casp8 WT cells (**Figures 1J, K**), which corresponded with worse OS (**Figures 1L, M**). EMT was characterized as a prometastatic process in the human cancers (28, 29). It was observed that c-Src and caspase-8 was pivotal for EMT in lung adenocarcinoma (**Figures 1N, O**). It was of note that Y380A mutation in the holoprotein of caspase-8 attenuated the spontaneous metastasis and impaired EMT process in lung adenocarcinoma (**Figures 1G–K, M–O**). Collectively, tyrosine 380 of caspase-8 in the c-Src-caspase-8 interaction was pivotal for metastasis through EMT in lung adenocarcinoma.

### c-Src Was Overactivated in Lung Adenocarcinoma Dependently on Caspase-8

**Figure 2:**
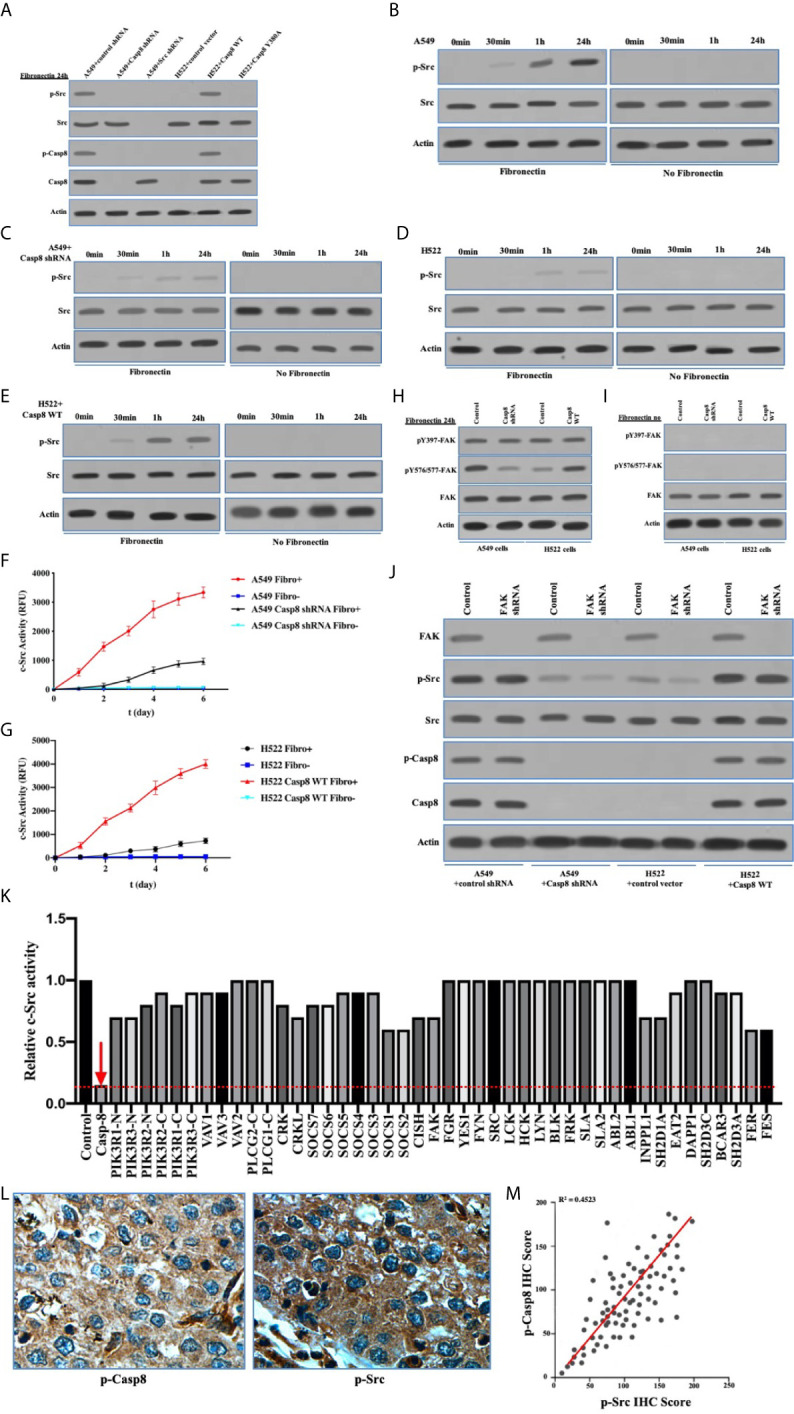
c-Src was overactivated in lung adenocarcinoma dependently on Caspase-8. **(A)** Immunoblotting analysis of p-Src, c-Src, p-Casp8, Caspase-8 and β-actin in A549 + control/Src/Casp8 shRNA and H522 + control vector/Casp8 WT/Casp8 Y380A attached on fibronectin for 24 h. **(B–E)** Immunoblotting analysis of p-Src, c-Src and β-actin in A549 + control **(B)** /Casp8 shRNA **(C)** and H522 + control vector **(D)** /Casp8 WT **(E)** attached on fibronectin or not for 24 h. **(F, G)** A549 cells with a lentiviral delivery of either control shRNA **(F)** or Casp8 shRNA and H522 cells with an adenoviral delivery of either control vector or Casp8 WT **(G)** attached on fibronectin or not were in the presence of a c-Src-specific fluorescent substrate, and fluorescence was recorded as the function of time (*p < 0.05). **(H, I)** Immunoblotting analysis of pY397-FAK, pY567/577-FAK, FAK and β-actin in A549 + control/Casp8 shRNA and H522 + control vector/Casp8 WT attached on fibronectin **(H)** or not **(I)** for 24 h. **(J)** Immunoblotting analysis of FAK, p-Src, c-Src, p-Casp8, Caspase-8 and β-actin in A549 + control/Casp8 shRNA and H522 + control vector/Casp8 WT with or without FAK knockdown attached on fibronectin for 24 h. **(K)** c-Src activity analysis in A549 cells with a set of siRNAs for targets (**Table S1**) as indicated attached on fibronectin for 24 h. **(L)** Positive expressions of p-Casp8 and p-Src examined by immunohistochemistry (IHC) of specific antibody in cancerous tissue (×400). **(M)** Correlation between p-Casp8 and p-Src expression in cancerous tissues.

As a non-receptor tyrosine kinase, activated c-Src underpinned the EMT of cancer cells following the multiple cell signaling (30). Caspase-8 was phosphorylated on tyrosine 380 (Tyr-380) by c-Src following the attachment to fibronectin (**Figure 2A**), which was consistent with previous report (25). It was of importance that c-Src was overactivated in a caspase-8–dependent manner (**Figure 2A**). It had been proven that integrin α_V_β_3_ is expressed on the surfaces of A549 and H522 cells (data not shown). With the attachment to fibronectin, A549 and H522 cells expressing caspase-8 displayed c-Src overactivation relative to those lacking caspase-8 or c-Src (**Figures 2B–E**). Fibronectin was deemed necessary to induce caspase-8-dependent c-Src overactivation. In the consonance with immunoblottings, caspase-8 knockdown or deficiency remarkably dampened c-Src activity in A549 and H522 cells attached to fibronectin (**Figures 2F, G**). It was intriguing that the basic activity of c-Src was maintained in A549 and H522 cells with caspase-8 knockdown or deficiency following fibronectin attachment (**Figures 2C, D, F, G**). This indicated that the extracellular stimuli maintained the basic activity of c-Src that initiated c-Src–caspase-8 interaction, while caspase-8 virtually overactivated c-Src in lung adenocarcinoma.

To confirm the specific role of caspase-8 on c-Src overactivation relative to other intracellular c-Src activators, such as focal adhesion kinase (FAK), we tested the interaction between c-Src and FAK, which has been reported to contribute to c-Src activation in various human tumors (31). It has also been reported that fibronectin-integrin triggered the autophosphorylation of Tyr 397 of FAK to facilitate c-Src activation, while Tyr 576/577 of FAK was phosphorylated by activated c-Src (30, 31). Autophosphorylation of Tyr 397 of FAK was dependent on fibronectin rather than on caspase-8, which was consistent. Caspase-8 knockdown or deficiency attenuated Tyr 576/577 phosphorylation of FAK (**Figures 2H, I**). We then knocked down FAK in A549 and H522 cells. It was of note that FAK phosphorylation was completely eliminated without fibronectin attachment (**Figure 2I**). Surprisingly, FAK knockdown slightly decreased c-Src activation in the caspase-8–lacking A549 and H522 cells (**Figure 2J**), while it was unable to remarkably affect caspase-8 phosphorylation and c-Src overactivation in the caspase-8-expressing A549 and H522 cells (**Figure 2J**). To further confirm the role of caspase-8 on c-Src overactivation in lung adenocarcinoma, we profiled a set of the potential activator of c-Src through the siRNA library (**Table S1**). Accordingly, caspase-8 was strongly associated with c-Src overactivation in lung adenocarcinoma (**Table S2** and **Figure 2K**). We retrospectively examined the association between p-Casp8 and p-Src in the patients with resectable lung adenocarcinoma (**Table 1**). Our examination revealed that p-Casp8 was positively correlated with p-Src in the lung adenocarcinoma tissues (**Figures 2L, M**). Together, caspase-8 was able to exclusively overactivate c-Src in lung adenocarcinoma.

**Table 1:** Association of phosphotyrosine 380 Caspase-8 (p-Casp8) expression with clinicopathological characteristics of patients with operable lung adenocarcinoma (n = 84).

Clinicopathological characteristics	p-Casp8 expression n (%)		*P*
	Positive	Negative	
Age (y)			
≤60	19 (42.2%)	16 (41.0%)	>0.05
>60	26 (57.8%)	23 (59.0%)	
Sex			
Male	35 (77.8%)	29 (74.3%)	>0.05
Female	10 (22.2%)	10 (25.7%)	
Differentiation			
Well	24 (53.3%)	23 (58.9%)	>0.05
Moderate	15 (33.3%)	13 (33.3%)	
Poor	6 (13.4%)	3 (7.8%)
Lymphnodes metastasis			
N_0-1_	22 (48.9%)	16 (41.0%)	>0.05
N_2-3_	23 (51.1%)	23 (59.0%)	
pTNM stage			
I-II	21 (46.7%)	17 (43.6%)	>0.05
III-IV	24 (53.3%)	22 (56.4%)	

### Phosphorylated Caspase-8 by c-Src Overactivated c-Src Through Its Phosphotyrosine 380 and “PDEP” Motif Docking to SH2 and SH3 Domain of c-Src

**Figure 3:**
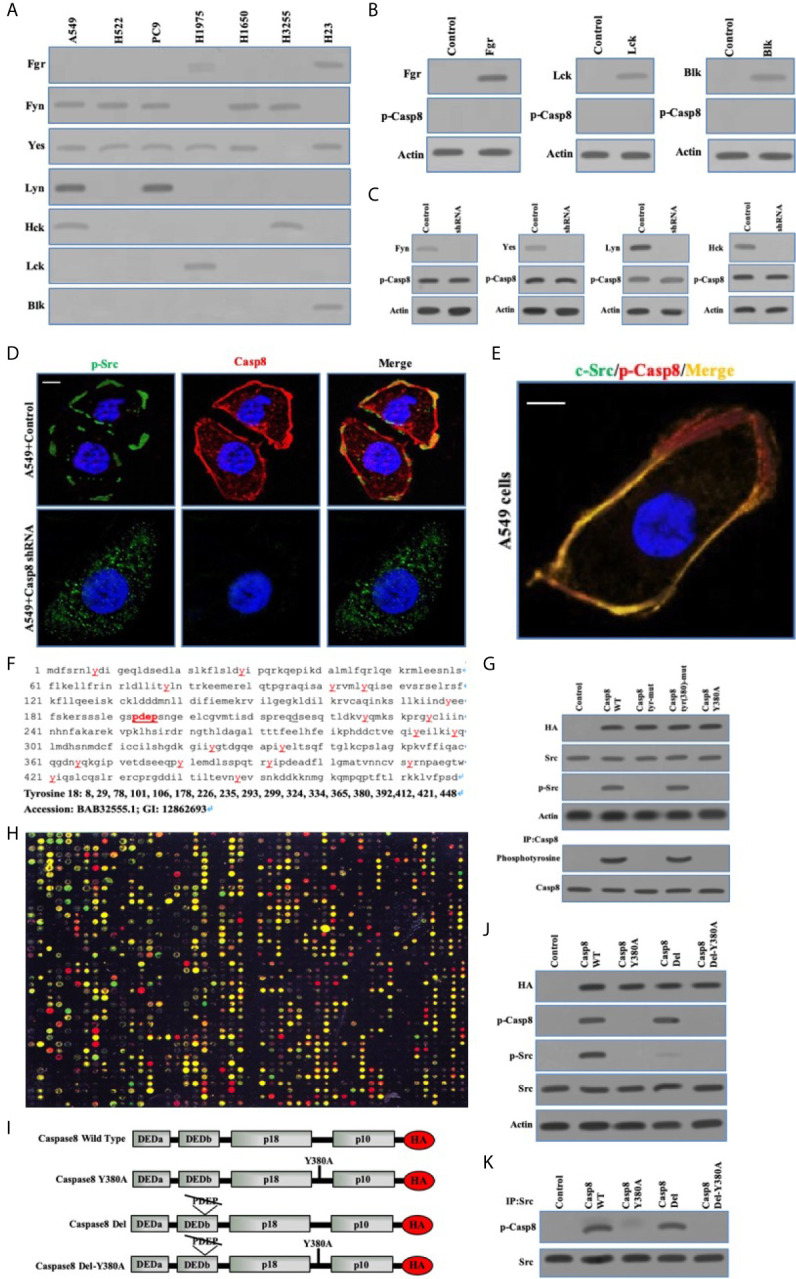
Phosphorylated Caspase-8 by c-Src overactivated c-Src through its phosphotyrosine 380 and “PDEP” motif docking to SH2 and SH3 domain of c-Src. **(A)** Immunoblotting analysis of Fgr, Fyn, Yes, Lyn, Hck, Lck and Blk in lung adenocarcinoma cell lines including A549, H522, PC9, H1975, H1650, H3255 and H23 cells attached on fibronectin for 24 h. **(B)** Immunoblotting analysis of Fgr, Lck, Blk and p-Casp8 in A549 cells transfected by adenoviral delivery of Fgr, Lck and Blk attached on fibronectin for 24 h. **(C)** Immunoblotting analysis of Fyn, Yes, Lyn, Hck and p-Casp8 in A549 cells transfected by lentiviral delivery of shRNAs of Fyn, Yes, Lyn and Hck attached on fibronectin for 24 h. **(D, E)** A549 cells were allowed to attach onto fibronectin-coated dish for 24 h and assessed by confocal microscopy using antibodies against p-Src, Casp8, c-Src and p-Casp8. Scale bars, 10 μm. **(F)** The amino acid sequence and tyrosine position of Caspase-8. **(G)** Immunoblotting analysis of HA, p-Casp8, c-Src, p-Src and β-actin in H522 cells with adenovirus encoding control vector, Casp8 WT, Casp8 tyr-mut, Casp8 tyr(380)-mut and Casp8 Y380A attached on fibronectin for 24 h. The TCLs of H522 cells with adenovirus encoding control vector, Casp8 WT, Casp8 tyr-mut, Casp8 tyr(380)-mut and Casp8 Y380A attached on fibronectin for 24 h were subjected to immunoprecipitation (IP) using anti-Caspase-8 antibody. The blot was then stripped and reprobed for phosphotyrosine and Casp8. **(H)** Microarray analysis of SH2 domains of intracellular proteins binding to p-Casp8 in A549 cells. Green and yellow represented equal signal and downregulation; red represented upregulation. **(I)** Schematic representation of various HA-tagged mutants of Caspase-8. **(J)** Immunoblotting analysis of HA, p-Src, p-Casp8, c-Src and β-actin in H522 cells with adenovirus encoding Caspase-8 mutants attached on fibronectin for 24 h. **(K)** The TCLs of H522 cells with adenovirus encoding Caspase-8 mutants in 3I were subjected to IP using anti-c-Src antibody. The blot was then stripped and reprobed for c-Src and p-Casp8.

To clarify the interplay between c-Src and caspase-8, we depicted the specificity of c-Src-induced caspase-8 phosphorylation and phosphorylated caspase-8–induced c-Src overactivation. Initially, we explored the expressions of other c-Src kinase family members in the lung adenocarcinoma cells. Fyn and Yes were frequently expressed in the lung adenocarcinoma cell lines (**Figure 3A**). The other c-Src kinase family members did not affect caspase-8 phosphorylation in A549 cells (**Figures 3B, C**). We applied immunofluorescence to reveal the subcellular locations of c-Src/caspase-8 and p-Src/p-Casp8. p-Src/caspase-8 and c-Src/p-Casp8 were co-localized in A549 cells (**Figures 3D, E**). There were 18 tyrosines in the holoprotein of caspase-8 (**Figure 3F**). We aimed to clarify whether tyrosine 380 of caspase-8 was specific for c-Src kinase substrate. Three mutants of caspase-8 were constructed, including caspase-8 with all tyrosine mutations (caspase-8 tyr-mut), all tyrosine mutations except for tyrosine 380 (caspase-8 tyr[380]-mut) and tyrosine 380 mutation (caspase-8 Y380A), all of which were stably transfected into H522 cells with caspase-8 deficiency. caspase-8 tyr(380)-mut and WT caspase-8 (caspase-8 WT) were able to similarly maintain caspase-8 tyrosine phosphorylation and c-Src overactivation instead of Casp8 Y380A (**Figure 3G**). This suggested that tyrosine 380 of caspase-8 was specific for c-Src-induced phosphorylation.

We explored the capability of the SH2 domains of human diverse proteins (**Table S1**) to bind to phosphotyrosine 380 of caspase-8. Phosphotyrosine 380 of caspase-8 docked to a huge number of human SH2 domains, including c-Src (**Figure 3H**), implying that the phosphotyrosine 380 site of caspase-8 was not specific for the SH2 domain of c-Src. A question was then raised as to how caspase-8 most potently overactivated c-Src in lung adenocarcinoma. Our team reanalyzed and scrutinized the sequence of caspase-8 holoprotein. The amino acid motif of “PDEP” (site 193-196) of caspase-8 might potentially bind to the SH3 domain (**Figure 3F**). A set of caspase-8 mutants as shown in **Figure 3I** were constructed and stably transfected into the caspase-8-lacking H522 cells by adenoviral vectors. Caspase-8 with “PDEP” motif deletion did not impair the phosphotyrosine 380 of caspase-8 in H522 cells (**Figure 3J**), whereas “PDEP”-deleted caspase-8 was unable to overactivate c-Src with no impacts on c-Src-induced caspase-8 phosphorylation (**Figure 3J**). The “PDEP” motif of caspase-8 was not required for its interaction with c-Src as tyrosine 380 of c-Src (**Figure 3K**). Collectively, caspase-8 phosphorylated by c-Src reversely overactivated c-Src through its phosphotyrosine 380 and “PDEP” motif docking to the SH2 and SH3 domains of c-Src, respectively.

### c-Src Was Unable to Trigger EGFR-Related Signaling Reflected by the Phosphotyrosines 1068, 1086, and 1145 of EGFR

**Figure 4:**
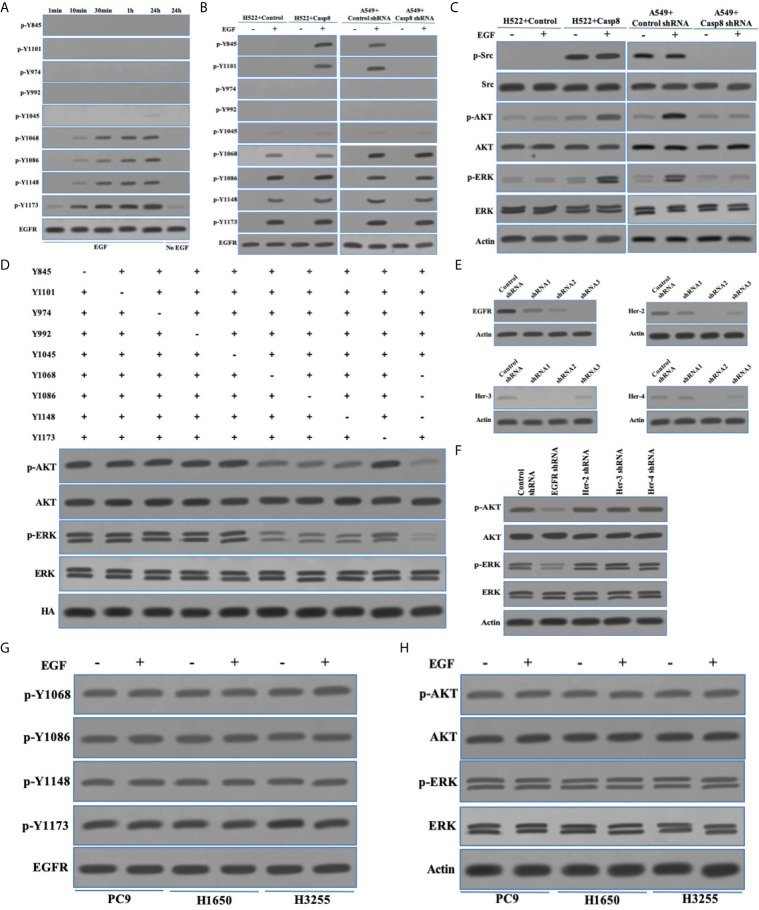
c-Src was unable to trigger EGFR-related signaling reflected by the phosphotyrosines 1068, 1086, and 1145 of EGFR. **(A)** Immunoblotting analysis of p-Y845, p-Y1101, p-Y947, p-Y992, p-Y1045, p-Y1068, p-Y1086, p-Y1148, p-Y1173 of EGFR and EGFR in H522 cells with Casp8 WT attached on fibronectin for 24 h stimulated by EGF or not as indicated times. **(B)** Immunoblotting analysis of p-Y845, p-Y1101, p-Y947, p-Y992, p-Y1045, p-Y1068, p-Y1086, p-Y1148, p-Y1173 of EGFR and EGFR in H522 cells with control vector or Casp8 WT and A549 cells with control shRNA or Casp8 shRNA attached on fibronectin for 24 h stimulated by EGF or not for 24 h. **(C)** Immunoblotting analysis of p-Src, c-Src, p-AKT, AKT, p-ERK, ERK and β-actin in H522 cells with control vector or Casp8 WT and A549 cells with control shRNA or Casp8 shRNA attached on fibronectin for 24 h stimulated by EGF or not for 24 h. **(D)** Immunoblotting analysis of p-AKT, AKT, p-ERK, ERK and HA in EGFR-deleted A549 cells transfected with lentiviral tyrosine mutants of EGFR attached on fibronectin for 24 h stimulated by EGF for 24 h. **(E)** Immunoblotting analysis of EGFR, Her-2, Her-3, Her-4 and β-actin in A549 cells with lentiviral shRNAs specific for control, EGFR, Her-2, Her-3 and Her-4. **(F)** Immunoblotting analysis of p-AKT, AKT, p-ERK, ERK and β-actin in A549 cells with lentiviral shRNAs specific for control, EGFR, Her-2, Her-3 and Her-4 attached on fibronectin for 24 h stimulated by EGF for 24 hr. **(G)** Immunoblotting analysis of p-Y1068, p-Y1086, p-Y1148, p-Y1173 of EGFR and EGFR in PC9, H1650 and H3255 cells stimulated by EGF or not for 24 hr. **(H)** Immunoblotting analysis of p-AKT, AKT, p-ERK, ERK and β-actin in PC9, H1650 and H3255 cells attached on fibronectin for 24 h stimulated by EGF or not for 24 hr.

EGFR signaling was critical to maintain proliferation and metastasis of NSCLC in particular for EGFR-mutant NSCLC (32, 33). The interaction between EGFR and other tyrosine kinases amplified the survival signaling through the phosphoinositide-3 kinase (PI3K)-protein kinase B (AKT) and the mitogen-activated protein kinase (MAPK)-extracellular signal-regulated kinase (ERK1/2 or ERK) pathway (32–34). Therefore, we hoped to uncover the relationship between c-Src activation and EGFR signaling in lung adenocarcinoma. We showed the activation model of EGFR in the EGFR-nonaddictive H522 cells lacking c-Src activation, in which the autophosphorylation of EGFR at tyrosine 1173 was paralleled with the phosphorylated tyrosine 1068, 1086, and 1148 with the epidermal growth factor (EGF) addition (**Figure 4A**), while the phosphorylation of tyrosine 845, 1101, 974, and 992 was undetectable (**Figure 4A**). It seemed that the tyrosine 1173 was more sensitive to reflect EGF-triggered EGFR activation (**Figure 4A**). EGFR activation was completely eliminated with no EGF addition in H522 cells (**Figure 4A**). In addition, tyrosine 1045 was slightly phosphorylated following EGF stimulation (**Figures 4A, B**). It was explicable that the phosphotyrosine 1045 initiated the ubiquitination and degradation of EGFR (35). It was of interest to clarify whether c-Src overactivation was able to affect EGFR activation in lung adenocarcinoma. In the EGFR-nonaddictive H522 cells and A549 cells with caspase-8 expression, the phosphorylation of tyrosine 845 and 1101 was significantly increased under the stimulation of EGF (**Figure 4B**), suggesting that the phosphorylation of tyrosine 845 and 1101 was associated with c-Src overactivation and EGF stimulation. EGFR activation depended on EGF in the EGFR-nonaddictive lung adenocarcinoma. EGF triggered the EGFR-associated downstream signaling including PI3K-AKT and MAPK-ERK (**Figure 4C**). It was noteworthy that EGF triggered survival signaling and did not affect c-Src activity in lung adenocarcinoma cells (**Figure 4C**).

We next evaluated the potency of phosphorylated tyrosine sites of EGFR to trigger the EGFR-related signaling. Our team constructed the tyrosine site mutants (tyrosine [Y] to alanine [A]) of EGFR tagged with HA tag in the EGFR-deleted A549 cells (**Figure 4D**). Accordingly, tyrosine 1068, 1086, and 1148 rather than tyrosine 1173 had the synergic effect to trigger PI3K-AKT and MAPK-ERK signaling (**Figure 4D**). It was of notes that c-Src activation led to two tyrosine sites (tyrosine 845 and 1101) of EGFR being phosphorylated and did not contribute to EGFR-related signaling. A previous study uncovered that EGFR with sensitive mutation was self-active through the form of homodimerization to trigger survival signaling (36). To explore the EGFR activation through EGFR homo- or hetero-multimerization, we synthesized shRNAs for targets on Her-2, Her-3, and Her-4 (**Figure 4E**). The knockdown of Her-2, Her-3, or Her-4 was incapable of affecting EGFR-related signaling as EGFR knockdown in the EGFR-nonaddictive lung adenocarcinoma cells (**Figure 4F**). This indicated that EGFR homodimerization might trigger EGFR-related signaling. We extended the molecular machinery for EGFR-related signaling in the EGFR-addictive lung adenocarcinoma cells with the sensitive mutation of EGFR (PC9 and H1650 with exon 19 deletion mutation [deletion E746-A750], H3255 with exon 21 L858R). In PC9, H1650, and H3255 cells, EGFR activation and EGFR-related signaling were similar regardless of EGF stimulation (**Figures 4G, H**). Together, the phosphorylation of tyrosine 1068, 1086, and 1148 was reflective of the EGFR activation accompanied by the EGFR-related signaling in lung adenocarcinoma.

### TKIs Blocked EGFR-Related Signaling to Facilitate Cell deaths of EGFR-Mutant Lung Adenocarcinoma

**Figure 5:**
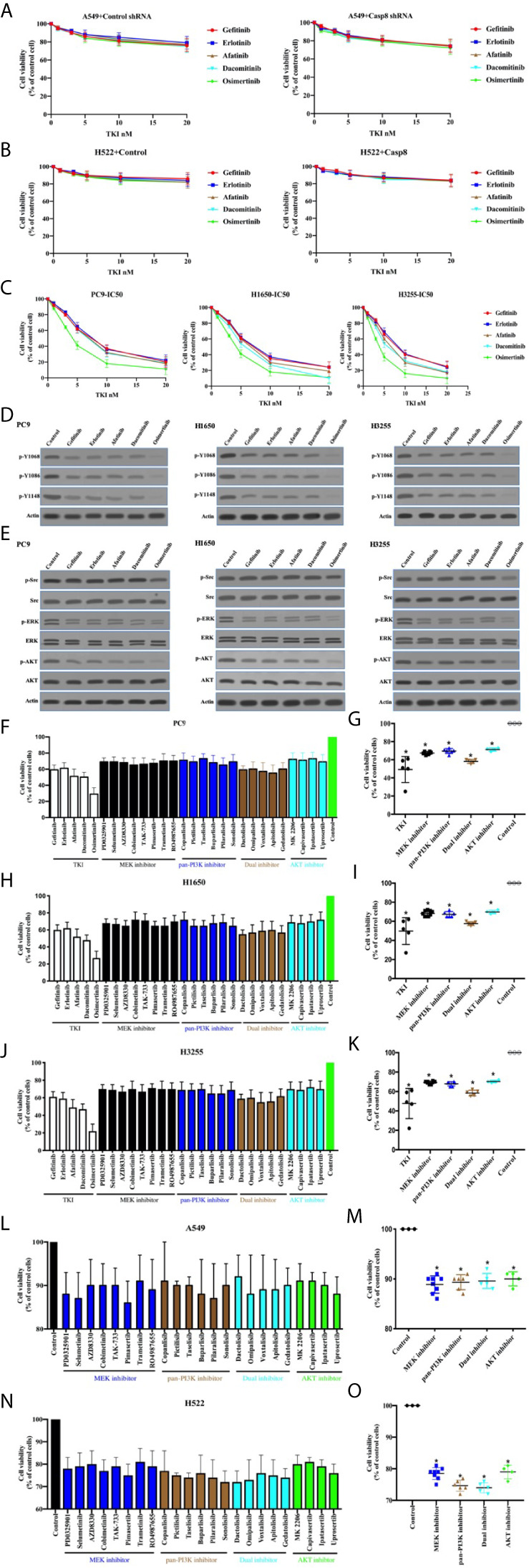
TKIs blocked EGFR-related signaling to facilitate cell deaths of EGFR-mutant lung adenocarcinoma. **(A)** Viability assay of A549 cells with control shRNA or Casp8 shRNA attached on fibronectin for 24 h treated by TKIs at indicated concentrations for 48 h (*n* = 3). **(B)** Viability assay of H522 cells with control vector or Casp8 WT attached on fibronectin for 24 h treated by TKIs at indicated concentrations for 48 h (*n* = 3). **(C)** Viability assay of PC9, H1650 and H3255 cells treated by TKIs at indicated concentrations for 48 h (*n* = 3). **(D)** Immunoblotting analysis of p-Y1068, p-Y1086, p-Y1148 of EGFR and β-actin in PC9, H1650 and H3255 cells. **(E)** Immunoblotting analysis of p-Src, c-Src, p-AKT, AKT, p-ERK, ERK and β-actin in PC9, H1650 and H3255 cells. **(F, G)** Viability assay of PC9 cells treated by drugs indicated for 48 h (*n* = 3). * *vs.* control, *p* < 0.05. **(H, I)** Viability assay of H1650 cells treated by drugs indicated for 48 h (*n* = 3). * *vs.* control, *p* < 0.05. **(J, K)** Viability assay of H3255 cells treated by drugs indicated for 48 h (*n* = 3). * *vs.* control, *p* < 0.05. **(L, M)** Viability assay of A549 cells attached on fibronectin for 24 h treated by drugs indicated for 48 h (*n* = 3). * *vs.* control, *p* < 0.05. **(N, O)** Viability assay of H522 cells attached on fibronectin for 24 h treated by drugs indicated for 48 h (*n* = 3). * *vs.* control, *p* < 0.05.

Targeted therapy with TKIs is characterized as a standard treatment for patients with EGFR-mutated lung adenocarcinoma (37, 38). It was deemed necessary to determine the antitumor effects and molecular mechanisms of TKIs in lung adenocarcinoma. As shown in **Figures 5A, B**, TKIs did not lead to the marked decrease in tumor cell viability in the EGFR-nonaddictive A549 and H522 cells with or without c-Src overactivation. By contrast, PC9, H1650, and H3255 cells with the sensitive mutation of EGFR showed the potent antitumor activity corresponding to distinct TKIs (**Figure 5C**). TKIs had the great efficacy to inhibit the phosphorylation of tyrosine 1068, 1086, and 1145 of EGFR in the EGFR-mutant lung adenocarcinoma cells (**Figure 5D**). In turn, TKIs efficiently suppressed PI3K-AKT and MAPK-ERK signaling, particularly for osimertinib (**Figure 5E**). It was intriguing that osimertinib could attenuate c-Src activation to a lesser extent (**Figure 5E**). Together, this indicated that TKIs induced the antitumor activity through the inhibition of EGFR-related signaling in the EGFR-mutant lung adenocarcinoma cells.

Sequentially, we sought to disclose the association between the inhibitions of PI3K-AKT/MAPK-ERK signaling and cell deaths. Our team procured a set of the inhibitors for PI3K-AKT and MAPK-ERK signaling (MAPK/ERK kinase; MEK) as shown in **Table S3**. In the EGFR-mutant cell lines, the variable inhibitors of PI3K-AKT and MAPK-ERK presented variable antitumor activity (**Figures 5F, H, J**), indicating that the ability of TKIs to eliminate tumor cells accounted for the inhibitor of survival signaling, including PI3K-AKT and MAPK-ERK. Nevertheless, PI3K-AKT and MAPK-ERK inhibition was unable to kill tumor cells as TKIs did (**Figures 5G, I, K**). This implied the possibility that the applied inhibitors at the concentration could not inhibit the survival signaling as TKIs alone did. We then attempted to clarify the effects of the survival signaling blockade on the viability of EGFR-nonaddictive cell lines. It was intriguing that the PI3K-AKT and MAPK-ERK blockade induced A549 and H522 cell deaths (**Figures 5L–O**). It seemed that H522 cells with caspase-8 deficiency were more sensitive to the inhibition of survival signaling (**Figures 5L, N**). Therefore, it was rationalized that TKIs could induce to antitumor activity in the EGFR-mutant lung adenocarcinoma cells through the inhibition of survival signaling.

### The Necroptosis Through FADD Complex Was Predominant for TKIs-Induced Cell Death in the EGFR-Mutant Lung Adenocarcinoma

**Figure 6:**
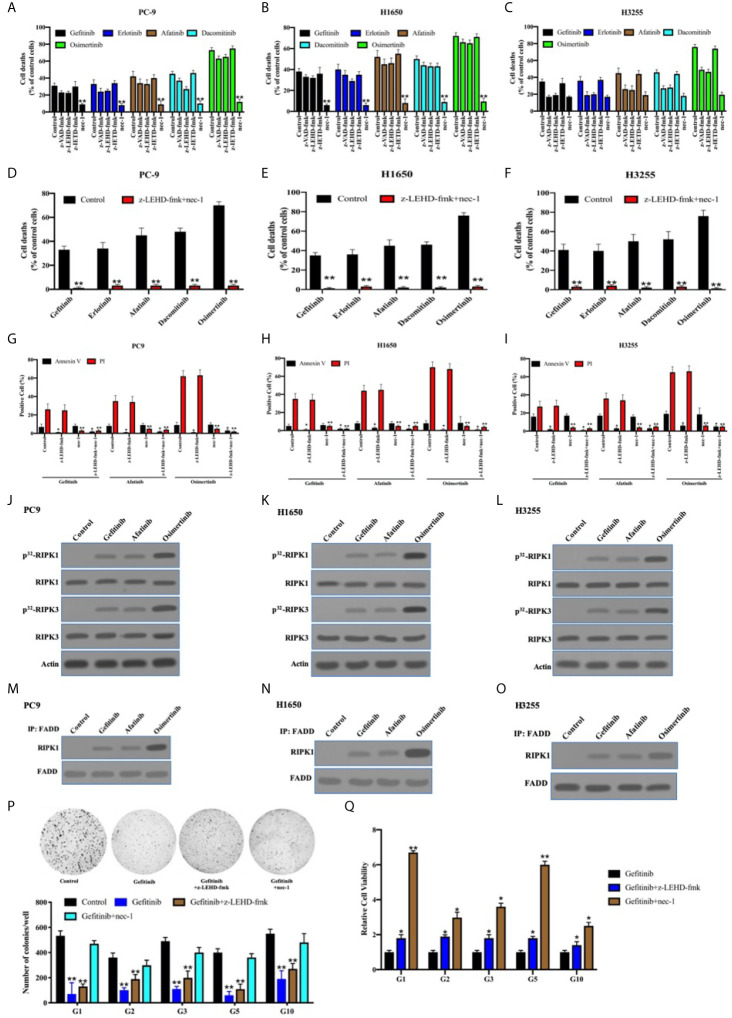
The necroptosis through FADD complex was predominant for TKIs-induced cell death in the EGFR-mutant lung adenocarcinoma. **(A–C)**, PC9, H1650, and H3255 cells were treated with TKIs with the addition of DMSO (Control), zIETD-fmk, zVAD-fmk, and nec-1 for 48 h. Cell viability was determined by measuring ATP levels using Cell Titer-Glo kit. Data were represented as mean ± standard deviation of duplicates. **, *p* < 0.01. **(D–F)** PC9, H1650, and H3255 cells were treated with TKIs with the addition of DMSO (Control) and zIETD-fmk+nec-1 for 48 h. Cell viability was determined by measuring ATP levels using Cell Titer-Glo kit. Data were represented as mean ± standard deviation of duplicates. ** *vs.* control, *p* < 0.01. **(G–I),** PC9, H1650, and H3255 cells were treated with TKIs with the addition of DMSO (Control), zIETD-fmk, nec-1 and zIETD-fmk+nec-1 for 48 h. Cells were analyzed for Annexin V/PI staining by flow cytometry. All experiments were repeated 3 times with similar results. **, *vs.* Control, *p* < 0.01. **(J–L)**, Immunoblotting analysis of RIPK1, RIPK3 and β-actin in PC9, H1650 and H3255 cells treated with TKIs 48 h. Cells were labelled with [^32^P]-orthophosphate. Phosphorylated RIPK1 and RIPK3 were measured by Cyclone Plus Phosphor Imager. **(M–O)** The immunocomplexes of PC9, H1650 and H3255 cells treated with TKIs for 48 h were eluted with antibody against FADD, and whole elution was used to measure RIPK1. **(P)** Soft agar colony formation assay of patient-derived tumor cells with DMSO (Control), gefitinib, gefitinib+zIETD-fmk and gefitinib+nec-1 in 12-well dish (5×10^3^ cells per well) for 1 weeks (*n* = 3). Representative images (*upper*) and average number of colonies (*lower*) are shown. **, *vs.* Control, *p* < 0.01. **(Q)** PC9, H1650, and H3255 cells were treated with gefitinib, gefitinib+zIETD-fmk and gefitinib+nec-1 for 48 h. Cell viability was determined by measuring ATP levels using Cell Titer-Glo kit. Data were represented as mean ± standard deviation of duplicates. * *vs.* control, *p* < 0.05. ** *vs.* control, *p* < 0.01.

It has been reported that TKIs induced apoptosis through the inhibition of survival pathways in EGFR-mutant lung adenocarcinoma (39, 40). We previously reported that necroptosis was predominant for chemotherapy-induced cell death in lung adenocarcinoma cells, owing to c-Src-induced caspase-8 phosphorylation to block apoptosis (21). In line with this, the inhibitor for caspase-8–induced apoptosis (z-IETD-fmk) did not impact the antitumor activity of TKIs in PC9, H1650, and H3255 cells expressing p-Casp8 (**Figures 6A–C**). We found that caspase-9 inhibitor (intrinsic apoptosis inhibitor: z-LEHD-fmk) and pan-caspase inhibitor (z-VAD-fmk) reduced approximately 8%, 18%, and 4% (4%–18%) of cell deaths in the EGFR-sensitive lung adenocarcinoma cells, while necrostatin-1 (necroptosis inhibitor; nec-1) salvaged the most cell deaths in lung adenocarcinoma (**Figures 6A–C**). To further dissect the models of TKI-induced cell death, flow cytometry analysis *via* Annexin V-propidium iodide (PI) staining was applied. Dual inhibitors for intrinsic apoptosis and necroptosis completely rescued the cell deaths of EGFR-mutated lung adenocarcinoma cells (Annexin V-positive: apoptosis; PI-positive: necroptosis; **Figures 6D–F**). This indicated that necroptosis was predominant in the TKI-induced cell deaths of lung adenocarcinoma, whereas the intrinsic apoptosis was highly variable. Caspase-9 inhibitor and nec-1 were able to prevent tumor cells from apoptosis and necroptosis, respectively, in the EGFR-mutant lung adenocarcinoma cells (**Figures 6G–I**).

It was shown that receptor-interacting serine/threonine-protein kinase (RIPK) 1 and RIPK3 were obviously phosphorylated in PC9, H1650, and H325 cells treated with TKIs (**Figures 6J–L**). It was previously reported that Fas-associated death domain (FADD) complex was the crux to balance between caspase-8–induced apoptosis and RIPK1-induced necroptosis (41–43). In line with that, FADD was coimmunoprecipitated with sharply increased RIPK1 under the stimulation of TKIs (**Figures 6M–O**). Our team harvested 33 pairs of EGFR-mutant lung adenocarcinoma and noncancerous tissues, of which 13 pairs were accessible to patient-derived xenografts (PDXs) or tumors. We sought to determine the cell death pattern in the patient-derived tumor cells with the sensitive-mutation EGFR (**Table S4**). The necroptosis accompanied by apoptotic inhibition contributed to a greater portion of TKI-induced cell deaths as compared with intrinsic apoptosis alone (**Figures 6P, Q**). This, therefore, indicated that the necroptosis was critical for TKI-induced antitumor activity in the EGFR-mutant lung adenocarcinomas.

### Inactivated c-Src Blocked EGFR-Related Survival Pathway Through Sealing the Phosphotyrosines of EGFR

**Figure 7:**
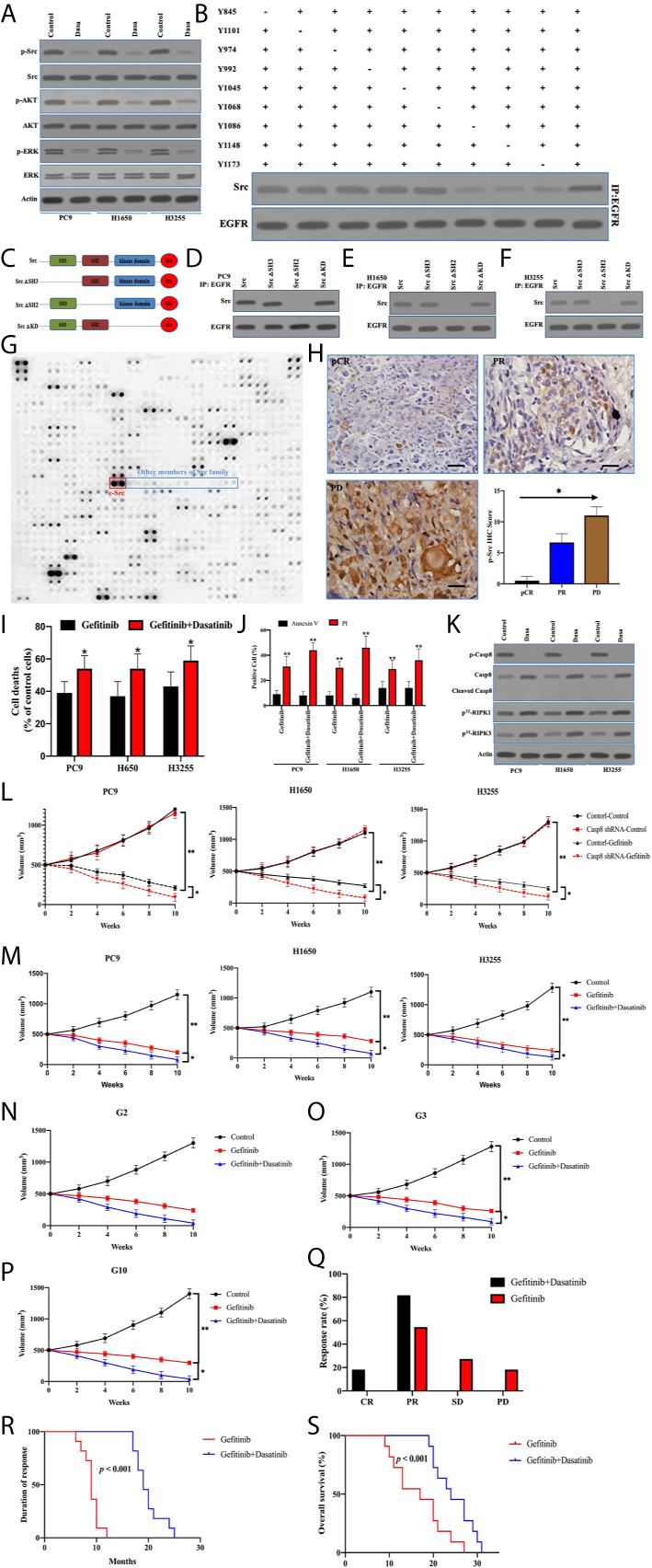
Inactivated c-Src blocked EGFR-related survival pathways through sealing the phosphotyrosines of EGFR triggering cell deaths. **(A)** Immunoblotting analysis of Caspase-8, p-Src, c-Src, p-AKT, AKT, p-ERK, ERK, and β-actin in PC9, H1650, and H3255 cells treated with control and dasatinib for 48 h. **(B)** The immunocomplexes of EGFR-deficient A549 cells transfected the adenoviral HA-tagged tyrosine mutants of EGFR treated with EGF were eluted with antibody against EGFR, and whole elution was used to measure c-Src. **(C)** Schematic representation of various mutants of c-Src used in this study. **(D–F)** The immunocomplexes of PC9, H1650, and H3255 cells transfected lentiviral Casp8 shRNA were eluted with antibody against EGFR, and whole elutions were used to measure HA. **(G)** Microarray analysis of SH2 domains of intracellular proteins binding to EGFR in A549 cells attached on fibronectin for 24 h. **(H)** Immunohistochemistry for p-Src expression in patients with EGFR-mutant lung adenocarcinoma representing CR, PR, and PD to TKIs by specific antibody (×400). The relationship between p-Src IHC score and chemotherapy response was showed. * vs. intergroup, *p* < 0.05. **(I)** PC9, H1650, and H3255 cells were treated with gefitinib or gefitinib+dasatinib for 48 h. Cell viability was determined by measuring ATP levels using Cell Titer-Glo kit. Data were represented as mean ± standard deviation of duplicates. * vs. Gefitinib, *p* < 0.05. **(J)** PC9, H1650, and H3255 cells were treated with gefitinib or gefitinib+dasatinib for 48 h. Cells were analyzed for Annexin V/PI staining by flow cytometry. All experiments were repeated 3 times with similar results. **, vs. Gefitinib, *p* < 0.01. **(K)** Immunoblotting analysis of Caspase-8, p-Casp8, cleaved Casp8, and β-actin in PC9, H1650 and H3255 cells attached on fibronectin for 24h treated with gefitinib+control (Control) and gefitinib+dasatinib (Dasa). Cells were labelled with [^32^P]-orthophosphate. Phosphorylated RIPK1, RIPK3 and MLKL were measured by Cyclone Plus Phosphor Imager. **(L)** Subcutaneous xenograft assay of PC9, H1650, and H3255 cells transfected with adenoviral control shRNA and Casp8 shRNA in nude mice treated with control or dasatinib. Volumes of tumors were shown (*n* = 10 per group). **p < 0.01. *p < 0.05. **(M)** Subcutaneous xenograft assay of PC9, H1650 and H3255 cells in nude mice treated with control or gefitinib or gefitinib+dasatinib. Volumes of tumors were shown (*n* = 10 per group). **, *p* < 0.01. *, *p* < 0.05. **(N–P)** Representative patient-derived tumor xenografts in nude mice treated with control or gefitinib or gefitinib+dasatinib. Volumes of tumors were shown (*n* = 10 per group). **p < 0.01. *p < 0.05. **(Q)** Response to gefitinib or gefitinib+dasatinib of patients with metastatic EGFR-mutant lung adenocarcinoma. **p < 0.01. **(R, S)** Kaplan-Meier analysis of duration of response and overall survival in the cohort of PDX models, *p* < 0.001.

Although activated c-Src did not enhance EGFR-related survival pathways in lung adenocarcinoma, the possibility of c-Src inactivation resulting in caspase-8 dephosphorylation to facilitate cell death was considered. p-Src and p-Casp8 were ubiquitous in EGFR-mutant lung adenocarcinomas (**Figure 5E** and **Table S4**). It was expected that c-Src inactivation was attained by dasatinib in the EGFR-mutant lung adenocarcinoma cells (21). The addition of dasatinib in the EGFR-mutant lung adenocarcinoma cells efficiently attenuated c-Src activation (**Figure 7A**). It was of note that c-Src inactivation had the potential to suppress EGFR-related survival pathways (**Figure 7A**). We then explored the interaction between c-Src and EGFR through the coimmunoprecipitation assay. Coimmunoprecipitated c-Src was remarkably reduced with the mutations of tyrosine 1068, 1086, and 1145 of EGFR in the EGFR-lacking A549 cells (**Figure 7B**), while the interplay between c-Src and EGFR was not affected in the other mutants of EGFR (**Figure 7B**). It was more likely that c-Src was binding to phosphotyrosine 1068, 1086, and 1145 to block the EGFR-induced survival pathways. Then, we constructed various c-Src mutants (**Figure 7C**). Moreover, the deletion of the SH2 domain of c-Src completely eliminated its interaction with EGFR in the EGFR-mutant lung adenocarcinoma (**Figures 7D–F**). We constructed the microarray of SH2 domains of intracellular proteins in the lung adenocarcinoma according to **Table S1**. Our data showed that a huge number of SH2 domains could bind to phosphorylated tyrosine 1068, 1086, and 1145 of EGFR, in which the SH2 domain of c-Src could more effectively bind to the phosphotyrosines of EGFR (**Figure 7G**). It seemed that other members of the c-Src family were not binding to the phosphotyrosines of EGFR as c-Src did (**Figure 7G**). We analyzed the correlation between c-Src activation and the therapeutic effect. It was intriguing that c-Src inactivation promoted the clinical response of TKIs (**Figure 7H**).

To answer the question of whether dasatinib facilitated caspase-8–induced apoptosis through caspase-8 dephosphorylation, dasatinib obviously increased TKI-induced cell deaths in PC9, H1650, and H3255 cells (**Figure 7I**). Strikingly, apoptosis was not significantly increased, compared with necrosis (**Figure 7J**). Dasatinib inactivated c-Src to dephosphorylate caspase-8 without the apoptotic cleavage of caspase-8 (**Figures 7A, K**), whereas RIPK1/RIPK3 phosphorylation was obviously enhanced (**Figure 7K**). This suggested that dasatinib promoted the necroptosis of TKIs through RIPK1/RIPK3 activation. On the other hand, caspase-8 knockdown with the impairment of c-Src activation significantly increased the response of the xenografts to gefitinib (**Figure 7L**), whereas dasatinib achieved a similar effect (**Figure 7M**). In the sequence, we observed the response of human lung adenocarcinoma with EGFR mutation to gefitinib and dasatinib in the PDX model. The addition of dasatinib consistently had the marked impacts on promoting the response of the EGFR-mutant lung adenocarcinoma to gefitinib (**Figures 7N–P**). Furthermore, our team retrospectively analyzed all the patients with EGFR-mutant lung adenocarcinoma in our center. 18 patients with metastatic lung adenocarcinoma who received gefitinib/erlotinib plus dasatinib were screened out. Of these patients, 10 received no treatment except for the combined treatment in **Table S5**. It was daunting that all eligible lung adenocarcinomas were p-Src-positive (**Table S5**). After being compared with the results of the EGFR-mutant lung adenocarcinoma patients treated by gefitinib alone, dual drugs of gefitinib and dasatinib achieved the better response rate and duration as compared with gefitinib alone (**Figures 7Q, R**), which led to a better prognosis for OS (**Figure 7S**). In sum, inactivated c-Src by dasatinib sealed the phosphorylated tyrosine 1068, 1086, and 1145 of EGFR to inhibit the survival pathway to sensitize EGFR-mutant lung adenocarcinoma to TKI.

## Discussion

NSCLC is the most commonly diagnosed human cancer as well as the leading cause of cancer-related deaths in 2019, of which lung adenocarcinoma accounted for 55% with a significant increase (3). The clinical utility of using a single gene-based biomarker as a therapeutic focus for lung adenocarcinoma was first realized with the discovery of mutations in the tyrosine kinase domain of EGFR in 2004; this enabled the identification of patients with greater sensitivity to TKIs (38, 44). The first-generation EGFR TKIs, gefitinib and erlotinib, designed to reversibly compete for the adenosine triphosphate binding sites and, thus, block EGFR-induced downstream signaling activation in the lung adenocarcinoma treatment (38, 45). A layer of complexity in EGFR signaling is the potential for cross-talk with cytoplasmic tyrosine kinases, particularly the ubiquitously expressed kinases c-Src (46). Therefore, we expected to uncover a novel path to sensitize TKIs in EGFR-mutant lung adenocarcinomas.

To date, there is increasing striking evidence that supports the nonapoptotic roles of caspase-8 (14, 47). This was substantiated by our observation that caspase-8 was rarely lacking in lung adenocarcinoma (21, 25). The linkage between caspase-8 and c-Src has been confirmed by the observation that cell stimulations with the survival-promoting factors led to c-Src-mediated caspase-8 phosphorylation on Tyr-380, and thus inhibiting its apoptotic function (18, 25). It was more likely that c-Src–caspase-8 interaction had a key role in human cancers. Therefore, we further dissected the interaction between c-Src and caspase-8 in lung adenocarcinoma cell lines. Strikingly, only tyrosine 380 out of 18 tyrosines of caspase-8 was clarified to be phosphorylated by activated c-Src. On the other hand, phosphorylated caspase-8 was the most powerful in inducing c-Src overactivation as compared with other putative activators of c-Src, such as EGFR and FAK. To answer the question as to why caspase-8 can furiously overactivate c-Src and surpass other putative activators, prior studies uncovered that c-Src was activated by docking to the SH2 domain or the SH3 domain with a higher affinity (35, 48). Caspase-8 had the phosphotyrosine 380 docking to the SH2 domain of c-Src and “PDEP” motif binding to the SH3 domain of c-Src to overactivate c-Src in lung adenocarcinoma. It was reasonable that EGFR and FAK could not activate c-Src due to a lack of binding to the SH3 domain of c-Src despite its phosphorylated tyrosines docking to the SH2 domain of c-Src.

EMT is a trans-differentiation characterized as a key step toward cancer metastasis with decreased epithelial markers, such as E-cadherin, and increased mesenchymal markers, such as vimentin (49, 50). Activated c-Src has been identified as a potent inducer for EMT (49, 50). Following phosphotyrosine 380 of caspase-8–mediated c-Src overactivation, we detected the downregulation of E-cadherin and upregulation of vimentin with morphological characteristics of spindle and dendritic shapes that could promote tumor metastasis in lung adenocarcinoma. Our previous data demonstrated that RNF43 was able to ubiquitinate and degrade E-cadherin phosphorylated by c-Src to facilitate the EMT process in lung adenocarcinoma, indicating the mechanism that activated c-Src induced the EMT phenotype (25). c-Src overactivation by caspase-8 triggered EMT to facilitate tumor metastasis and yielded resistance to therapies in lung adenocarcinoma. Hence, the blockade of caspase-8-induced c-Src overactivation shed a light on this topic in clinical practice.

EGFR is a receptor tyrosine kinase that is frequently upregulated in human cancers, such as in NSCLC (51, 52). Diverse mechanisms augmented EGFR activity, including the EGFRvIII truncations, as well as to its kinase domain, such as the L858R mutations (52). These EGFR aberrations overactivated downstream pro-oncogenic signaling pathways, including the MAPK-ERK and PI3K-AKT pathways (51). These survival pathways activated many biological outputs that were beneficial to cancer cell proliferation. Nevertheless, the EGFR activation model remained elusive. Tyrosine 1068, 1086, and 1143 of EGFR were characterized to reflect EGFR activation in lung adenocarcinoma, and were irreplaceable for EGFR-related signaling. The notion has been supported by previous reports that tyrosine 1068, 1086, and 1143 of EGFR played a dominant role on EGFR activation in lung cancer (40). Our observation differed from a previous study in that EGFR was homo- or heteromultimerized with Her-2, Her-3, and Her-4. Her-2 was an important effector that leads to the resistance of TKIs to tumor cells. We found that Her-2, Her-3, or Her-4 had less contribution to EGFR activation. It was inferred that EGFR homomultimerization was critical for EGFR activation.

Surprisingly, EGFR signaling was different in the capability to maintain tumor growth in the different lung adenocarcinoma cells. The EGFR-mutation lung adenocarcinomas were more addictive to EGFR-induced survival signaling than the EGFR-WT lung adenocarcinomas. TKIs efficiently blocked EGFR-induced PI3K-AKT and MAPK-ERK in order to initiate necroptosis to the majority and intrinsic apoptosis to a lesser extent. This implied that EGFR addiction for tumor growth might underlie the clinical value of TKIs. Accordingly, intrinsic apoptosis was highly variable in the TKI-induced cell deaths of EGFR-mutant lung adenocarcinoma. This may be because the intrinsic apoptotic pathway was too entangled to disentangle, involving too many molecular factors. Dauntingly, dasatinib inactivated c-Src to seal the survival signaling-related phosphorylated tyrosines of EGFR by the SH2 domain of c-Src to facilitate necroptosis instead of caspase-8–induced apoptosis. Our team has done great work in exploring why caspase-8 dephosphorylation could not initiate apoptosis during dasatinib treatment in lung adenocarcinoma (data unpublished). However, dasatinib had limited benefit for advanced EGFR-mutant NSCLC (53, 54). It was more likely that dasatinib was unable to efficiently inactivate c-Src kinase to maintain its clinical therapeutic value.

**Figure 8:**
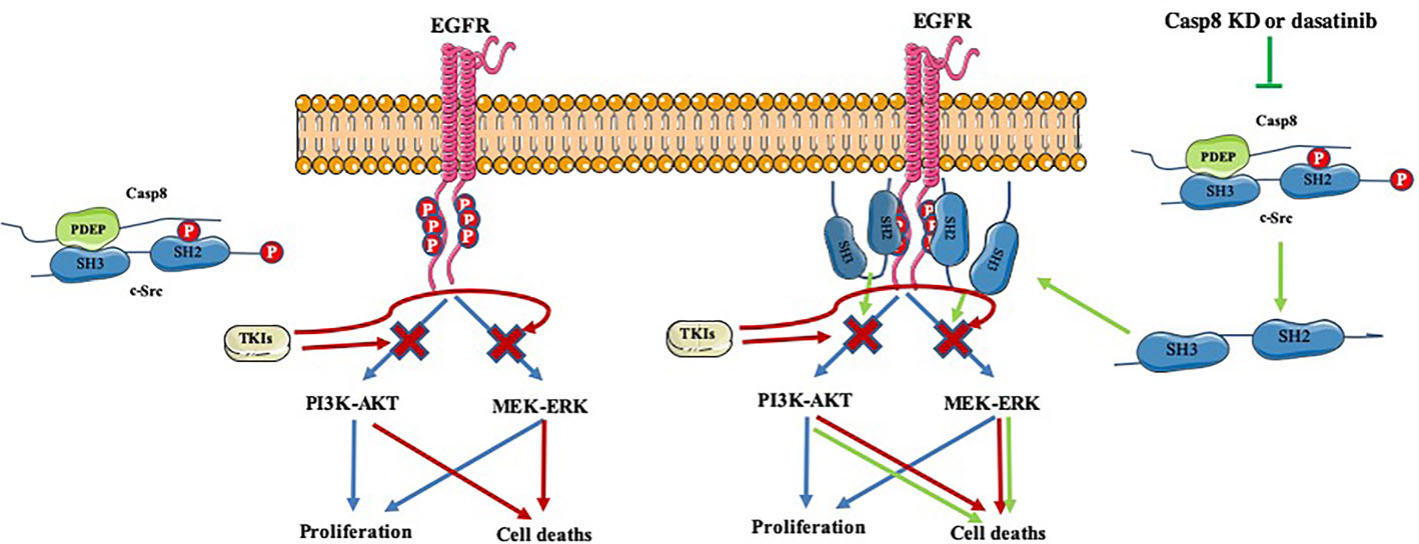
A schematic diagram of synergic effects of inactivated c-Src and TKIs.

Collectively, caspase-8 phosphorylated at tyrosine 380 by c-Src predominantly enhanced c-Src activation to induce EMT phenotypic features in lung adenocarcinoma. Mechanistically, the linkage of the c-Src SH2 domain with phosphotyrosine 380 of caspase-8 and SH3 domain with “PDEP” motif of caspase-8 furiously overactivated c-Src. In addition, activated EGFR reflected by the levels of phosphotyrosine 1068, 1086, and 1145 of EGFR had no impact on c-Src activation, while TKIs attenuated EGFR activation to induce cell deaths of lung adenocarcinoma. Surprisingly, blocking c-Src activation through dasatinib was able to inhibit the EGFR survival signaling by sealing tyrosine 1068, 1086, and 1145 of EGFR, which in turn increased the antitumor activity of TKIs in EGFR-mutant lung adenocarcinoma. Together, inactivated c-Src by dasatinib administration sensitized EGFR-mutant lung adenocarcinoma to TKIs (**Figure 8**).

## Data Availability Statement

The original contributions presented in the study are included in the article/**Supplementary Materials**. Further inquiries can be directed to the corresponding author.

## Ethics Statement

The studies involving human participants were reviewed and approved by the Ethics Committee of the Second Affiliated Hospital of Xi’an Jiaotong University. Written informed consent to participate in this study was provided by the participants’ legal guardian/next of kin. The animal study was reviewed and approved by the Ethics Committee of the Second Affiliated Hospital of Xi’an Jiaotong University. Written informed consent was obtained from the owners for the participation of their animals in this study. Written informed consent was obtained from the individual(s), and minor(s)’ legal guardian/next of kin, for the publication of any potentially identifiable images or data included in this article.

## Author Contributions

Conception and design: YZ. Administrative support: SZ. Provision of study materials or patients: YZ and CH. Collection and assembly of data: YZ, WM, and CH. Manuscript writing: YZ. Final approval of manuscript: YZ. All authors contributed to the article and approved the submitted version.

## Funding

Supported by National Natural Science Foundation of China (No. 81301847 and No. 81872390).

## Conflict of Interest

The authors declare that the research was conducted in the absence of any commercial or financial relationships that could be construed as a potential conflict of interest.

## Glossary

**Table:**

EMT	Epithelial-mesenchymal transition
Tyr	Tyrosine
NSCLC	Non–small cell lung cancer
WT	Wild type
EGFR	Epidermal growth factor receptor
PI3K	Phosphoinositide-3 kinase
AKT	Protein kinase B
MAPK	Mitogen-activated protein kinase
ERK1/2 or ERK	Extracellular signal-regulated kinase
MEK	MAPK/ERK kinase
FAK	Focal adhesion kinase
HA	Hemagglutinin
TKI	Tyrosine kinase inhibitor
PDX	Patient-derived xenografts
CR	Complete remission
PR	Partial remission
SD	Stable disease
PD	Progressive disease
AJCC	American Joint Committee on Cancer
RECIST	Response evaluation criteria in solid tumors
IHC	Immunohistochemistry
NCI	National cancer institute
ATCC	American type culture collection
FBS	Fetal bovine serum
shRNAs	Short hairpin RNAs
Ct	Threshold cycle
nec-1	Necrostatin-1
IP	Immunoprecipitation
PI	Propidium iodide
IHC	Immunohistochemistry
RIPK	Receptor-interacting serine/threonine-protein kinase
FADD	Fas-associated death domain
ARMS	Amplification refractory mutation system
