# Supplementary material for: c-Src Increases the Sensitivity to TKIs in the EGFR-Mutant Lung Adenocarcinoma
Source: Front Oncol. 2021 Jul 22;11:602900. doi: 10.3389/fonc.2021.602900 (PMC8339729; doi:10.3389/fonc.2021.602900)
Supplement: Supplementary file 1 [file DataSheet_1.docx]

**Supplementary materials**

**Materials and Methods**

**Reagents and drugs**

TKIs (gefitinib, erlotinib, afatinib, dacomitinib and osimertinib) and inhibitors for PI3K-AKT and MAPK-ERK were purchased from MedChemExpress for cell culture and animal studies at 4°C at concentrations of 1 M at origin. The concentrations of TKIs was applied based on IC50. The inhibitors for PI3K-AKT and MAPK-ERK were applied at the concentrations as shown in Table S3. EGF was purchased (0.1 mg/ml) from Invitrogen and applied at concentration of 4 pg/ml. TKIs were applied at 10 nM. Dasatinib (Sprycel, Bristol-Myers Squibb) was dissolved in dimethyl sulfoxide (DMSO) and used at a final concentration of 10^-6^ M. z-VAD-fmk (50 mM), z-LEHD-fmk (50 mM) and z-IETD-fmk (50 mM) were obtained from Alexis Biochemicals. Necrostatin-1 (nec-1, 10 mM) was generously gifted by Chen Huang from the Department of Cell Biology, Xi’an Jiaotong University, Shaanxi Province, P.R. China.

**EMT signaling**

Cells were serum-starved for 24 hr and plated onto 100 mm non-tissue culture-treated dishes (1.0 × 10^6^ per dish) precoated with 10 μg/ml fibronectin (Millipore). The morphological changes were observed following 5-day attachment on the fibronectin-coated dishes. EMT-related signaling were analyzed after 24 hr attachment on the fibronectin-coated dishes. At each time point, adherent cells were directly lysed. Cells lysates were analyzed by immunoblotting against the specific antibodies as described.

**Immunohistochemistry**

After routine deparaffinization and hydration, tissue sections were treated with 3% hydrogen peroxide and heated in EDTA (pH 8.0) for antigen retrieval. Following serum blocking, p-Casp8 and p-Src antigen-antibody reactions took place at 4 °C overnight. The streptavidin/peroxidase kit (Invitrogen) was used to detect antigen-antibody reactions. The purified rabbit/rat monoclonal antibodies against human p-Casp8 and p-Src were used at 2 μg/ml and goat anti-rabbit/rat biotin-conjugated IgG was used as secondary antibody. Immunohistochemical signals were scored by two independent observers. The scores were calculated as the number of stained cells divided by the total number of cancer cells counted. Four high-power fields (× 400) per slide were calculated, then the results were averaged. Unequivocal staining of cytoplasm in > 50% of cancer cells was considered as positive.

The updated H-score method (p-Casp8/p-Src IHC by H-score with magnification rule), first developed was recently adapted(Bankhead et al., 2018). This method assigns an IHC H-score to each patient on a continuous scale of 0–300, based on the percentage of cells at different staining intensities visualized at different magnifications. Membrane staining was scored according to four categories: 0 for ‘no staining’, 1 + for ‘light staining visible only at high magnification’, 2 + for ‘intermediate staining’ and 3 + for ‘dark staining of linear membrane, visible even at low magnification’. The percentage of cells at different staining intensities was determined by visual assessment, with the score calculated using the formula 1 × (% of 1+ cells) + 2 × (% of 2+ cells) + 3 × (% of 3+ cells). The outcome-based discriminatory threshold IHC H-score for this analysis was set at 200 and existing samples were re-read and scored according to the above method. Samples were then classified as either low (H-score < 200; IHC negative) or high (≥200; IHC positive) for protein expression.

**Immunoblotting and immunoprecipitation**

Protein extracts were resolved on 10% SDS-polyacrylamide gels (SDS-PAGE) and electrophoretically transferred onto polyvinylidene difluoride (PVDF, Millipore) membranes. Membranes were blocked for 1 hr in TBST [20 mmol/L Tris-HCl (pH 7.6), 150 mmol/L NaCl, 0.05% Tween] containing 5% non-fat dried milk or 5% bovine serum albumin (BSA, Sigma-Aldrich), then were incubated rocking at 4 °C overnight with appropriate primary antibodies: anti-Src (sc-130124, 1:500; Santa Cruz Biotechnology), anti-p-Src (ab-4816, 1:20000; Abcam), anti-Caspase-8 and cleaved Caspase-8 (sc-81656, 1:600; Santa Cruz Biotechnology); anti-p-Casp8 (PA5-39716 1:500; ThermoFisher Scientific); anti-RIPK1 (ab-72139, 1:2000; Abcam); anti-RIPK3 (ab-152130, 1:1000; Abcam); anti–β-actin (sc-130300, 1:1000; Santa Cruz Biotechnology); anti-hemagglutinin (anti-HA; 12CA5, 1:2000 Roche Applied Science); anti-FADD (ab-24533, 1:10000; Abcam); anti-E-cadherin (ab-1416, 1:1000; Abcam); anti-Vimentin (ab-8978, 1:700; Abcam); anti-EGFR (ab-52894, 1:2000; Abcam); anti-phophotyrosine (ab-179530, 1:1000; Abcam); anti-pY845-EGFR (ab-109685, 1:3000; Abcam); anti-pY974-EGFR (ab-228993, 1:2000; Abcam); anti-pY992-EGFR (ab-81440, 1:1000; Abcam); anti-pY1045-EGFR (ab-24928, 1:1000; Abcam); anti-pY1101-EGFR (ab-76195, 1:1000; Abcam); anti-pY1068-EGFR (ab-40815, 1:5000; Abcam); anti-pY1086-EGFR (ab-32086, 1:5000; Abcam); anti-pY1148-EGFR (ab-135560, 1:1000; Abcam); anti-pY1173-EGFR (ab-32578, 1:5000; Abcam); anti-Fgr (ab-133349, 1:5000; Abcam); anti-Fyn (ab-125016, 1:5000; Abcam); anti-Yes (ab-109265, 1:5000; Abcam); anti-Lyn (ab-1890, 1:2000; Abcam); anti-Hck (ab-61055, 1:2000; Abcam); anti-Lck (ab-32149, 1:6000; Abcam); anti-Blk (ab-189320, 1:4000; Abcam); anti-AKT (AKT1/2/3, ab-16798, 1:5000; Abcam); anti-p-AKT (S473, ab-81283, 1:5000; Abcam); anti-ERK (ERK1/2, ab-17942, 1:5000; Abcam); anti-p-ERK (T202/Y204, ab-223500, 1:5000; Abcam); anti-FAK (ab-40794, 1:1000; Abcam); anti-pY397-FAK (ab-81298, 1:8000; Abcam); anti-pY576/577-FAK (ab-76244, 1:8000; Abcam). After incubation for 1 hr with anti-rabbit/rat secondary antibodies conjugated to horseradish peroxidase (Jackson ImmunoResearch Laboratories Inc.), bands were visualized using chemiluminescence (SuperSignal West Pico Chemiluminescent substrate, Pierce). For immunoprecipitation, 500 μg protein was incubated with 2 μg specific antibody. Immunocomplexes were precipitated with 25 μl protein A/G (Pierce) or glutathione (GE Healthcare) beads. Then, beads were washed three times, eluted in the boiling Laemmli buffer, resolved on 10% SDS-PAGE and electrophoretically transferred onto PVDF membranes as described above. Protein samples for dimerization analysis were prepared under non-reducing condition. Then, the cell lysates were resolved on non-reducing gel and analyzed by immunoblotting with anti-FADD or anti-HA antibody.

**Metabolic labelling with [^32^P]-phosphate**

10^6^ cells were resuspended at 1.0 ml of phosphate-free medium and incubated for 40 min at 37 °C. Cells were spun down and resuspended in phosphate-free medium containing 1 mCi of [^32^P]-orthophosphate and incubated for 2 hr prior to agents or drugs stimulations. Immunoprecipitations were performed with RIPK1 or RIPK3-specific antibody and resolved on SDS-PAGE. [^32^P]-labeling phosphates were measured by Cyclone Plus Phosphor Imager (PerkinElmer).

**Immunofluorescence**

Cells were permitted to attach onto coverslips precoated with fibronectin (10 μg/mL) for 6 days, such that they were confluent and allowed to activate the cellular signaling. Subsequently, cells were fixed with 4% paraformaldehyde for 10 min, permeabilized in PBS containing 0.1% Triton X-100 for 3 min, and blocked for 60 min at room temperature with 2% BSA in PBS. Cells were then stained with monoclonal antibody to E-cadherin (1:100, Abcam), Vimentin (1:100, Abcam), Caspase-8 (1:100; ThermoFisher Scientific) c-Src (1:100; Abcam) and p-Src (1:100; Abcam) for 1 hr. After washing several times in PBS, cells were exposed to secondary antibody specific for rat (Alexa 488 or 565, 1:300; Invitrogen). Samples were mounted in Vectashield hard set mounting medium (Vector Laboratories) and imaged on a Nikon Eclipse C1 confocal microscopy.

**siRNA library screening**

As reported before(Zheng et al., 2014), 1×10^5^ A549 cells were seeded in 24-well plates, 18 hr prior to transfection. On the following day, 10ng/ml final concentration of pooled siRNAs (3 siRNAs/gene) were transfected using RNAiMAX reagent (Life Technology). Cells were collected 48 hr post-transfection and assayed for luciferase activity using the Glomax 96 microplate luminometer (Promega).

**Cell viability assay**

Cell viability was evaluated as previously reported(Degterev et al., 2005). We seeded cells in 96-well plates (white plates for luminescent assays; black plates for fluorescent assays; clear plates for MTT assays) at the density of 5,000-10,000 cells per well for adherent cells in 100 ml of appropriate phenol red-free media. After incubation, we determined cell viability using one of the following methods. For the ATP assay, we used luminescence-based commercial kits (CellTiter-Glo, Promega or ATPLite-M, PerkinElmer) and analyzed luminescence using a Wallac Victor II plate reader (PerkinElmer). For Sytox assay, we incubated cells with 1 mmol/L Sytox Green reagent for 30 min at 37 °C, and performed fluorescent reading. Subsequently, we added 5 ml of 20% Triton X-100 solution into each well to produce maximal lysis and incubated cells for 1 hr at 37 °C, then performed the second reading. We calculated the ratio of values (percentage of dead cells in each well) before and after Triton treatment and normalized it to the relevant controls not subjected to cytotoxic stimuli.

**Annexin V/propidium iodide (PI) assay**

Cells were seeded in 6-well plates at appropriate density and treated with the different sets of agents or drugs for 48 hr. Cells were then harvested and stained with Annexin V and PI and then analyzed on a cytofluorimeter by FACScan (BD Biosciences). PI-positive cells were considered as necrotic cells (necroptosis), Annexin V-positive/propidium iodide-negative as apoptotic cells. All untreated controls ranged from 5 % to 10 % of cell deaths. Results were expressed as ratios of the death cells: the total cells. For cell cycle assay, cells (1.0-2.0 × 10^6^ per sample) were harvested, and resuspended in 200 μl 1× PBS. The cells were fixed with 4 ml of cold 75 % ethanol at 4 °C for at least 4 hr and then washed twice with 1× PBS. Then the cells were resuspended in 500 μl 1× PBS and stained with 200 μl propidium iodide (50 μl/ml; Sigma-Aldrich) and 20 μl RNase (1 mg/mL; Sigma-Aldrich) in a 37 °C incubator for 15–20 min. Samples were determined by FAC Station (BD Biosciences) and analyzed by using CellQuest software. The assay was repeated three times.

**Real-time PCR for metastatic tumor cells**

Genomic DNA was extracted from harvested chick embryo lung tissues using the Puregene DNA purification system (Gentra Systems). To detect tumor cells in chick embryo lung tissues, primers specific for human alu sequences (sense: 5’ ACG CCT GTA ATC CCA GCA CTT 3’; antisense: 5’ TCG CCC AGG CTG GAG TGC A 3’) were used to amplify human alu repeats in genomic DNA extracted from chick embryo lung tissues. The real-time PCR used to amplify and detect alu sequence contained 30 ng of genomic DNA, 2 mM MgCl_2_, 0.4 μM each primer, 200 μM DNTP, 0.4 units of Platinum Taq polymerase (Invitrogen), and a 1:100000 dilution of SYBR green dye (TaKaRa). PCR was performed in a final volume of 10 μl under 10 μl of mineral oil with the iCycler iQ (Bio-Rad laboratories) under the following conditions: polymerase activation at 95 °C for 2 min followed by 30 cycles at 95 °C for 30 s, 63 °C for 30 s, and 72 °C for 30 s. A quantitative measure of amplifiable chick DNA was obtained through amplification of the chick GAPDH (chGAPDH) genomic DNA sequence with chGAPDH primers (sense: 5’ GAG GAA AGG TCG CCT GGT GGA TCG 3’; antisense: 5’ GGT GAG GAC AAG CAG TGA GGA ACG 3’) using the same real-time PCR conditions. The fluorescence emitted by the reporter dye was detected online, and the threshold cycle (Ct) of each sample was recorded as a quantitative measure of the amount of PCR product in the sample. The Ct was the cycle number at which the fluorescence generated by the reporter dye exceeded a fixed level above baseline. As indicated, the alu signal was normalized against the relative quantity of GAPDH and expressed as △Ct = (Ct_GAPDH_ - Ct_alu_). Each assay included a negative control, a positive control, a no-template control, and the experimental samples in duplicate.

**Tumor metastasis assay (*in vivo*)**

The assay was done as described in details by (Zijlstra et al., 2002). Cells (1.0 × 10^6^) resuspended in 40 μl serum-free medium were seeded onto the surface of chick embryo chorioallantoic membrane and assessing the presence of metastatic events in chick embryo lungs by amplification of a human-specific alu sequence as reported previously(Zijlstra et al., 2002). After tumor cells implantation for 10 days, tumors were resected and weighted.

**Spontaneous human xenograft metastasis**

Athymic BALB/c nude mice (4 weeks) were purchased from Shanghai experimental animal center and maintained in the experimental animal center of Xi’an Jiaotong University, in accordance with the university institutional animal care and use committee. Cells were cultured in fresh medium for 24 hr and harvested, adjusting cell concentration to 2.5 × 10^7^/ml with 50% matrigel (Sigma) in serum-free RPMI-1640 medium. A volume of 0.2 ml of the mixture was injected subcutaneously into the flank of mice. Xenograft growth was supervised (10/group) with dasatinib/gefitinib 25 mg/kg daily (via oral gavage) starting 20 days after tumor implantation. Weekly, mice were anesthetized with isofluorane (Janssen Pharmaceutica), injected i.p. with 225 mg/g body weight n-luciferin (potassium salt; Xenogen Corp.) and imaged on the IVIS-100 bioluminescence imager (Xenogen Corp.). As necessary, the mice were euthanatized by cervical dislocation with rapid unconsciousness. The national guidelines for the care and use of animals were followed along with the committee of Xi’an Jiaotong University.

**Patient-derived xenograft (PDX) and treatment experiments**

To establish patient-derived xenografts, primary tumor specimens were collected from lung adenocarcinoma patients who performed core needle biopsy at the Second Affiliated Hospital of Xi’an Jiaotong University (Shaanxi, China) between 2016 and 2017. The clinical features of patients were showed in Table S4. Eight-week-old athymic BALB/c nude mice under the pathogen-free conditions were used for patient-derived xenograft transplantation. Briefly, a small incision was made on the abdomen of anaesthetized BALB/c nudes and primary tumor samples were minced into 0.5 mm^3^ sized fragments and injected subcutaneously. The incision was then closed up with sutures. The time from cancer samples collection to mice implantation ranges from 30-180 min. The tumor formation was monitored in the next three months since implantation. As necessary, the mice were euthanatized by cervical dislocation with rapid unconsciousness. The national guidelines for the care and use of animals were followed along with the committee of Xi’an Jiaotong University.

For isolation of xenografted tumor cells, xenografts were mechanically disaggregated, washed with cold PBS containing penicillin (500 U/ml), streptomycin (500 μg/ml), gentamicin (100 mg/L) and amphotericin B (2.5 mg/L), and digested in DMEM/F12 medium containing type II/IV collagenase (1 mg/ml) and DNase (1 mg/ml) at 37 °C for 1 hr with intermittent shaking. The cell suspension was filtered through an 80 μm filter (BD Biosciences, USA) and centrifuged at 300 g for 5 min at 4°C. The pellets were suspended and seeded in 12-well plate.

**Peptide synthesis**

SH2 domain peptides were synthesized on the solid phase (50 mmol scale) using Fmoc chemistry according to previous report(Jones et al., 2006). Peptides were labelled on their amino termini with 5- (and-6)-carboxytetramethylrhodamine (5(6)-TAMRA) before deprotection and cleavage. All peptides were purified by reverse phase high-performance liquid chromatography.

**Fabrication and processing of microarrays**

Purified SH2 domains were spotted in duplicate at a concentration of 40 mM onto aldehyde- modified glass substrates (112.5 mm × 74.5 mm × 1 mm) using a piezoelectric microarrayer(Jones et al., 2006). Ninety-six identical arrays were fabricated in a 12 × 8 pattern to match the spacing of a microtitre plate. Each array consisted of a 14 × 14 pattern of spots, with a 250 mm pitch. After a 1 h incubation, the glass was attached to a bottomless 96-well plate using an intervening silicone gasket. Immediately before use, the plates were quenched with buffer B (20 mM HEPES, 100 mM KCl, 0.1% Tween-20, pH 7.8) containing 1% BSA (w/v). The proteins of tumor cells were extracted using a lysis buffer containing 10.0 nmol/L Tris (pH 7.4), 1.0 mmol/L sodium orthovanadate, 1.0% SDS. The lysate was heated and sonicated for 10-30 s. The protein concentration was determined with the protein assay kit (Bio-Rad, Hercules, CA). Protein extracts were frozen and sent on dry ice to BD Biosciences for analysis. The monoclonal anti-mouse antibodies against phosphorylated Caspase-8 (p-Casp8) and EGFR (p-Y1068, p-Y1086 and p-Y1148 EGFR) were used to create microarray. After incubation for 1 hr with anti-rat secondary antibodies conjugated to horseradish peroxidase (Jackson ImmunoResearch Laboratories Inc.), signals were visualized using chemiluminescence (SuperSignal West Pico Chemiluminescent substrate, Pierce). The chemiluminescence signals for each spot were captured analyzed using a microarray scanner (Axon Instruments Inc., Foster City, CA). Primary analysis was done using the Genepix software package. Images of scanned microarrays were gridded and linked to a protein print list. The method is highly specific and can detect subnanogram levels of protein.

**Statistical analysis**

SPSS 16.0 software was used to perform the statistical analysis. The experiments *in vitro* were performed at least thrice in triplicates. When the data from different groups were compared, normal analysis and homogeneity of variance were checked first, and then an unpaired two-tailed *t* test analysis was used. Error bars on the graphs as well as data in the text represented the mean ± SD. For *in vivo* studies, statistical power was realized by evaluating cohorts including all animals from all experiments using *χ^2^* and Mann-Whitney statistical tests. Pearson correlation analysis was performed to determine the correlation between two variables. Pearson’s chi-square test was used to analyze the clinical variables. Univariate survival analysis was carried out by Kaplan-Meier method, and subject to the log rank test. *P* < 0.05 was considered as significant.

**Supplementary references:**

Bankhead, P., Fernandez, J.A., McArt, D.G., Boyle, D.P., Li, G., Loughrey, M.B., et al. (2018). Integrated tumor identification and automated scoring minimizes pathologist involvement and provides new insights to key biomarkers in breast cancer. *Lab Invest* 98(1)**,** 15-26. doi: 10.1038/labinvest.2017.131.

Degterev, A., Huang, Z., Boyce, M., Li, Y., Jagtap, P., Mizushima, N., et al. (2005). Chemical inhibitor of nonapoptotic cell death with therapeutic potential for ischemic brain injury. *Nat Chem Biol* 1(2)**,** 112-119. doi: 10.1038/nchembio711.

Jones, R.B., Gordus, A., Krall, J.A., and MacBeath, G. (2006). A quantitative protein interaction network for the ErbB receptors using protein microarrays. *Nature* 439(7073)**,** 168-174. doi: 10.1038/nature04177.

Zheng, H., Shen, M., Zha, Y.L., Li, W., Wei, Y., Blanco, M.A., et al. (2014). PKD1 phosphorylation-dependent degradation of SNAIL by SCF-FBXO11 regulates epithelial-mesenchymal transition and metastasis. *Cancer Cell* 26(3)**,** 358-373. doi: 10.1016/j.ccr.2014.07.022.

Zijlstra, A., Mellor, R., Panzarella, G., Aimes, R.T., Hooper, J.D., Marchenko, N.D., et al. (2002). A quantitative analysis of rate-limiting steps in the metastatic cascade using human-specific real-time polymerase chain reaction. *Cancer Res* 62(23)**,** 7083-7092.
