# Supplementary material for: c-Src Increases the Sensitivity to TKIs in the EGFR-Mutant Lung Adenocarcinoma
Source: Front Oncol. 2021 Jul 22;11:602900. doi: 10.3389/fonc.2021.602900 (PMC8339729; doi:10.3389/fonc.2021.602900)
Supplement: Supplementary file 2 [file DataSheet_2.zip › Supplementary Table 1.DOCX]

**Table S1. Multiple sequences of human SH2 domains**

PIK3R1-N (1) --------GMNNNMSLQDAEWYWGDISRE---------EVNEKLRDTAD------GTFLVRDASTKMHG-DYTLTLRKGGNNK---------------LI

PIK3R3-N (1) --------MKDSSVSLQDAEWYWGDISRE---------EVNDKLRDMPD------GTFLVRDASTKMQG-DYTLTLRKGGNNK---------------LI

PIK3R2-N (1) --------NGGSPPSLQDAEWYWGDISRE---------EVNEKLRDTPD------GTFLVRDASSKIQG-EYTLTLRKGGNNK---------------LI

PIK3R2-C (1) --------DEDDLPHHEERTWYVGKINRT---------QAEEMLSGKRD------GTFLIRESS--QRG-CYACSVVVDGDTK---------------HC

PIK3R1-C (1) --------DDEDLPHHDEKTWNVGSSNRN---------KAENLLRGKRD------GTFLVRESS--KQG-CYACSVVVDGEVK---------------HC

PIK3R3-C (1) -------EEDENLPHYDEKTWFVEDINRV---------QAEDLLYGKPD------GAFLIRESS--KKG-CYACSVVADGEVK---------------HC

VAV1 (1) ---------HGPPQDLSVHLWYAGPMERA---------GAESILANRSD------GTFLVRQRV-KDAA-EFAISIKYNVEVK---------------HI

VAV3 (1) --------CVPKPVDYSCQPWYAGAMERL---------QAETELINRVN------STYLVRHRT-KESG-EYAISIKYNNEAK---------------HI

VAV2 (1) --------PPSREIDYTAYPWFAGNMERQ---------QTDNLLKSHAS------GTYLIRERP-AEAE-RFAISIKFNDEVK---------------HI

PLCG2-C (1) -------DPVPNPNPHESKPWYYDSLSRG---------EAEDMLMRIPRD-----GAFLIRK-REGSD--SYAITFRAR-----------G----KVKHC

PLCG1-C (1) --------PVPQTNAHESKEWYHASLTRA---------QAEHMLMRVPRD-----GAFLVRK-RNEPN--SYAISFRAE-----------G----KIKHC

CRK (1) --------MAGNFDSEERSSWYWGRLSR---------QEAVALLQGQ---RH---GVFLVRDSSTSPG--DYVLSVSENS---------------RVSHY

CRKL (1) -------MSSARFDSSDRSAWYMGPVSR---------QEAQTRLQGQ---RH---GMFLVRDSSTCPG--DYVLSVSENS---------------RVSHY

SOCS7 (1) ---------AASLRELEKCGWYWGPMNWE---------DAEMKLKGKPD------GSFLVRDSS-DPRY-ILSLSFRSQGITH---------------HT

SOCS6 (1) ---------TEELKKLAKQGWYWGPITRW---------EAEGKLANVPD------GSFLVRDSS-DDRY-LLSLSFRSHGKTL---------------HT

SOCS5 (1) ----------PDLLQITGNPCYWGVMDRY---------EAEALLEGKPE------GTFLLRDSA-QEDY-LFSVSFRRYNRSL---------------HA

SOCS4 (1) ----------PDLLQINNNPCYWGVMDKY---------AAEALLEGKPE------GTFLLRDSA-QEDY-LFSVSFRRYSRSL---------------HA

SOCS3 (1) ---------VNAVRKLQESGFYWSAVTGG---------EANLLLSAEP---A---GTFLIRDSS-DQRH-FFTLSVKTQSG---------------TKNL

SOCS1 (1) ----DYRRITRASALLDACGFYWGPLSVHG---------AHERLRAEP---V---GTFLVRDSR-QRNC-FFALSVKMASG---------------PTSI

SOCS2 (1) ----QAARLAKALRELGQTGWYWGSMTVN---------EAKEKLKEAPE------GTFLIRDSS-HSDY-LLTISVKTSAGPT---------------NL

CISH (1) --------IAKTFSYLRESGWYWGSITAS---------EARQHLQKMPE------GTFLVRDST-HPSY-LFTLSVKTTRGPT---------------NV

FGR (1) -----PSNYVAPVDSIQAEEWYFGKIGRK---------DAERQLLSPGNPQ----GAFLIRESETTKG--AYSLSIRDWDQ----------TRGDHVKHY

YES1 (1) --------YVAPADSIQAEEWYFGKMGRK---------DAERLLLNPGN-M----RYFLRKRDETTKG--AYSLSIRDWDE----------VRGDNVKHH

FYN (1) ----IPSNYVAPVDSIQAEEWYFGKLGRK---------DAERQLLSFGNPR----GTFLIRESETTKG--AYSLSIRDWDD----------MKGDHVKHY

SRC (1) ---YIPSNYVAPSDSIQAEEWYFGKITRR---------ESERLLLNAENPR----GTFLVRESETTKG--AYCLSVSDFDN----------AKGLNVKHY

LCK (1) -----PFNFVAKANSLEPEPWFFKNLSRK---------DAERQLLAPGNTH----GSFLIRESESTAG--SFSLSVRDFDQ----------NQGEVVKHY

HCK (1) ----------ARVDSLETEEWFFKGISRK---------DAERQLLAPGNML----GSFMIRDSETTKG--SYSLSVRDYDP----------RQGDTVKHY

LYN (1) -----PSNYVAKLNTLETEEWFFKDITRK---------DAERQLLAPGNSA----GAFLIRESETLKG--SFSLSVRDFDP----------VHGDVIKHY

BLK (1) --------FVARVESLEMERWFFRSQGRK---------EAERQLLAPINKA----GSFLIRESETNKG--AFSLSVKDVT-----------TQGELIKHY

FRK (1) --------YVAEDRSLQAEPWFFGAIGRS---------DAEKQLLYSENKT----GSFLIRESESQKG--EFSLSVLDGA---------------VVKHY

SLA (1) ----IPGICVARVYHG----WLFEGLGRD---------KAEELLQLPDTKV----GSFMIRESETKKG--FYSLSVRHR----------------QVKHY

SLA2 (1) ----IPSVHVAKVSHG----WLYEGLSRE---------KAEELLLLPGNPG----GAFLIRESQTRRG--SYSLSVRLSRP----------ASWDRIRHY

ABL2 (1) ----VPSNYITPVNSLEKHSWYHGPVSRS---------AAEYLLSSLIN------GSFLVRESESSPG--QLSISLRYEG---------------RVYHY

ABL1 (1) -----PSNYITPVNSLEKHSWYHGPVSRN---------AAEYLLSSGIN------GSFLVRESESSPG--QRSISLRYEG---------------RVYHY

INPPL1 (1) -------PGPGGALGSQAPSWYHRDLSR---------AAAEELLARAGR--D---GSFLVRDSESVAG--AFALCVLYQK---------------HVHTY

SH2D1A (1) ---------------MDAVAVYHGKISRE---------TGEKLLLATGLD-----GSYLLRDSESVPG--VYCLCVLYHG---------------YIYTY

EAT2 (1) ----------------MDLPYYHGRLTKQ---------DCETLLLKEGVD-----GNFLLRDSESIPG--VLCLCVSFKN---------------IVYTY

DAPP1 (1) -----WSRSDGEAELLQDLGWYHGNLTRH---------AAEALLLSNG--CD---GSYLLRDSNETTG--LYSLSVRAKDS---------------VKHF

SH2D3C (1) -------ELKLSSTDLRSHAWYHGRIPR---------EVSETLVQRN--------GDFLIRDSLTSLG--DYVLTCRWRN---------------QALHF

BCAR3 (1) -KKELEEELLLSSEDLRSHAWYHGRIPR---------QVSENLVQRD--------GDFLVRDSLSSPG--NFVLTCQWKN---------------LAQHF

SH2D3A (1) --------VPQDGEDLAGQPWYHGLLSRQ---------KAEALLQQN--------GDFLVRASGSRGG--NPVISCRWRG---------------SALHF

FER (1) ------DMISISEKPLAEQDWYHGAIPR---------IEAQELLKKQ--------GDFLVRESHGKPG--EYVLSVYSDG---------------QRRHF

FES (1) ----------EVQKPLHEQLWYHGAIPR---------AEVAELLVHS--------GDFLVRESQGKQ---EYVLSVLWDG---------------LPRHF

E105251 (1) ---------VDPALPLEKQPWFHGPLNRA---------DAESLLSLCKE------GSYLVRLSE-TNPQ-DCSLSLRSSQGFL---------------HL

SHB (1) ------GERVDPAVPLEKQIWYHGAISRG---------DAENLLRLCKE------CSYLVRNSQ-TSKH-DYSLSLRSNQGFM---------------HM

E138606 (1) --------WTDPALPLENQVWYHGAISRT---------DAENLLRLCKE------ASYLVRNSE-TSKN-DFSLSLKSSQGFM---------------HM

E169291 (1) ------GEKVDPGLPLEKQPWYHGAISRA---------EAESRLQPCKE------AGYLVRNSE-SGNS-RYSIALKTSQGCV---------------HI

SH2B (1) -----EPEGGEGDQPLSGYPWFHGMLSR---------LKAAQLVLTGGTGSH---GVFLVRQSETRRG--EYVLTFNFQG---------------KAKHL

APS (1) -------PEAEPELELSDYPWFHGTLSR---------VKAAQLVLAGGPRNH---GLFVIRQSETRPG--EYVLTFNFQGK-------------AKASHL

LNK (1) ----LDPACQKTDHFLSCYPWFHGPISR---------VKAAQLVQLQGPDAH---GVFLVRQSETRRG--EYVLTFNFQG---------------IAKHL

SHC1 (1) --------SVSMAEQLRGEPWFHGKLSR---------REAEALLQLN--------GDFLVRESTTTPG--QYVLTGLQSG---------------QPKHL

E185634 (1) --------LPHIKQQLWSEECYHGKLSR----------KAAESLLVKD-------GDFLVRESATSPG--QYVLSGLQGG---------------QAKHL

SHC3 (1) -------PDAKMLEELQAETWYQGEMSR---------KEAEGLLEKD--------GDFLVRKSTTNPG--SFVLTGMHNG---------------QAKHL

RASA1-N (1) -------EVAIPLTAPPTNQWYHGKLDRT---------IAEERLRQAGKS-----GSYLIRESDRRPG--SFVLSFLSQMN--------------VVNHF

PLCG1-N (1) ---------SSSTELHSNEKWFHGKLGAGRDGRHIAERLLTEYCIETG-APD---GSFLVRESETFVG--DYTLSFWRNG---------------KVQHC

PLCG2-N (1) --------DIPPTELHFGEKWFHKKVEK----RTSAEKLLQEYCMETG-GKD---GTFLVRESETFPN--DYTLSFWRSG---------------RVQHC

RASA1-C (1) --------VGREEDPHEGKIWFHGKISKQ---------EAYNLLMTVG--QV---CSFLVRPSDNTPG--DYSLYFRTNEN---------------IQRF

RIN3 (1) --------SILEKLIKTCPVWLQLSLGQA---------EVARILHRVVA------GMFLVRRDS-SSK----QLVLCVHFPSL----------NESSAEV

RIN2 (1) -------LSILDRLLHTHPIWLQLSLSEE---------EAAEVLQAQPP------GIFLVHKST-KMQ----KKVLSLRLP------------CEFGAPL

RIN1 (1) -----RVVSLRERLLLTRPVWLQLQANAA---------AALHMLRTEPP------GTFLVRKSNTRQCQ-ALCMRLPEASGPS-----------FVSSHY

TENC1 (1) ---TQESQSNVKFVQDTSKFWYKPHLSRD---------QAIALLKDKDP------GAFLIRDSH-SFQG-AYGLALKVATPPP---SAQPWKGDPVE---

TENS1 (1) -----------KFVQDTSKFWYKADISRE---------QAIAMLKDKEP------GSFIVRDSH-SFRG-AYGLAMKV----------------------

TNS (1) -------RAKVKFVQDTSKYWYKPEISRE---------QAIALLKDQEP------GAFIIRDSH-SFRG-AYGLAMKVSSPP------------------

CTEN (1) ---------TMKFVMDTSKYWFKPNITRE---------QAIELLRKEEP------GAFVIRDSS-SYRG-SFGLALKVQE--------------------

JAK1 (1) -------DVAPPLIVHNIQNGCHGPICTE---------YAINKLRQEGSEE----GMYVLRWSC-TDFD-NILMTVTCFEKSEQV-----QGAQKQFK--

JAK2 (1) ----------PPAVLENIQSNCHGPISMD---------FAISKLKKAGNQT----GLYVLRCSP-KDFN-KYFLTFAVEREN-----------VIEYKHC

JAK3 (1) -----CKEVAPPRLLEEVAEQCHGPITLD---------FAINKLKTGGSRP----GSYVLRRSP-QDFD-SFLLTVCVQNPL-----------GPDYKGC

BRDG1 (1) ----------DYVDVLNPMPACFYTVSRK---------EATEMLQKNPS-----LGNMILRPGSDSRN---YSITIRQEIDIP------------RIKHY

E109111 (1) ------------MKRKQQRTTYIKRVIAHPSFHNINFKQAEKMMETMDQ------GDVIIRPSSKGEN--HLTVTWKVSDG--------------IYQHV

STAT1 (1) --------IKKHLLPLWNDGCIMGFISKE---------RERALLKDQQP------GTFLLRFSESSREG-AITFTWVERSQNGG----------EPDFHA

STAT4 (1) ------DLIKKHILPLWIDGYVMGFVSKE---------KERLLLKDKMP------GTFLLRFSES-HLG-GITFTWVDHSESG-----------EVRFHS

STAT3 (1) -------LVKKYILALWNEGYIMGFISKE---------RERAILSTKPP------GTFLLRFSESSKEG-GVTFTWVEKDISG-----------KTQIQS

STAT2 (1) --------VHDHLKDLWNDGRIMGFVSRS---------QERRLLKKTMS------GTFLLRFSES-SEG-GITCSWVEHQDDD-----------KVLIYS

STAT5a (1) ----VMEVLKKHHKPHWNDGAILGFVNKQ---------QAHDLLINKPD------GTFLLRFSDS-EIG-GITIAWKFDSPER-----------N--LWN

STAT6 (1) ----VLDLTKRCLRSYWSDRLIIGFISKQ---------YVTSLLLNEPD------GTFLLRFSDS-EIG-GITIAHVIRGQDG-----------SPQIEN

CHN1 (1) -------TCEVENRPKYYGREFHGMISRE---------AADQLLIVAEG-------SYLIRESQRQPG--TYTLALRFGS---------------QTRNF

CHN2 (1) ----------VENRPKYYGREFHGIISRE---------QADELLGGVEG-------AYILRESQRQPG--CYTLALRFGN---------------QTLNY

PTK6 (1) ----VPHNYLAERETVESEPWFFGCISRS---------EAVRRLQAEGN-AT---GAFLIRVSE-KPSA-DYVLSVRDTQ---------------AVRHY

E18941 (1) --------LVESEGSLTENIWAFAGISRP------------CALALLRRDVL---GAFLLWPELGASG--QWCLSVRTQCG---------------VVPH

TEC (1) --------TGKKSNNLDQYEWYCRNMNRS---------KAEQLLRSEDKE-----GGFMVRDSS--QPG-LYTVSLYTKFGG---------EGSSGFRHY

BTK (1) ----PSNYVTEAEDSIEMYEWYSKHMTRS---------QAEQLLKQEGKE-----GGFIVRDSS--KAG-KYTVSVFAKSTG---------DPQGVIRHY

ITK (1) ---PSSYLVEKSPNNLETYEWYNKSISRD---------KAEKLLLDTGKE-----GAFMVRDSR--TAG-TYTVSVFTKAVVS--------ENNPCIKHY

TXK (1) --------TENKITNLEIYEWYHRNITRN---------QAEHLLRQESKE-----GAFIVRDSR--HLG-SYTISVFMGARR---------STEAAIKHY

BMX (1) --------SSEEEENLDDYDWFAGNISRS---------QSEQLLRQKGKE-----GAFMVRNSS--QVG-MYTVSLFSKAVN---------DKKGTVKHY

LCP2 (1) --------SPAEEENSLNEEWYVSYITRP---------EAEAALRKINQD-----GTFLVRDSSKKTTTNPYVLMVLYKD---------------KVYNI

MIST (1) ---RPPFPKRSDRKDVQHNEWYIGEYSRQ---------AVEEAFMKENK--D---GSFLVRDCSTKSKEEPYVLAVFYEN---------------KVYNV

BLNK (1) -------TISEQEAGVLCKPWYAGACDRK---------SAEEALHRSNK--D---GSFLIRKSSGHDSKQPYTLVVFFNKR---------------VYNI

SH3BP2 (1) --------GKPGLLQVPLPNSVFVNTTES--------CEVERLFKATSPRGEPQDGLYCIRNSSTKSGK---VLVVWDETSN-------------KVRNY

SYK-N (1) ------MASSGMADSANHLPFFFGNITRE---------EAEDYLVQGGMSD----GLYLLRQSRNYLG--GFALSVAHGR---------------KAHHY

ZAP70-N (1) -----------MPDPAAHLPFFYGSISRA---------EAEEHLKLAGMAD----GLFLLRQCLRSLG--GYVLSLVHDV---------------RFHHF

GRB14 (1) -----SQSSATNMAIHRSQPWFHHKISR---------DEAQRLIIQQG-LVD---GVFLVRDSQSNPK--TFVLSMSHGQ---------------KIKHF

GRB10 (1) --------STLSTVIHRTQHWFHGRISR---------EESHRIIKQQG-LVD---GLFLLRDSQSNPK--AFVLTLCHHQ---------------KIKNF

GRB7 (1) ------SGTSLSAAIHRTQLWFHGRISR---------EESQRLIGQQG-LVD---GLFLVRESQRNPQ--GFVLSLCHLQ---------------KVKHY

ZAP70-C (1) -SQAPQVEKLIATTAHERMPWYHSSLTRE---------EAERKLYSGAQTD----GKFLLR-PRKEQG--TYALSLIYGK---------------TVYHY

SYK-C (1) -SQKPQLEKLIATTAHEKMPWFHGKISRE---------ESEQIVLIGSKTN----GKFLIR-ARDNNG--SYALCLLHEG---------------KVLHY

MATK (1) ------REALSADPKLSLMPWFHGKISG---------QEAVQQLQPP---ED---GLFLVRESARHPG--DYVLCVSFGR---------------DVIHY

CSK (1) --------GVKAGTKLSLMPWFHGKITR---------EQAERLLYPP---ET---GLFLVRESTNYPG--DYTLCVSCDG---------------KVEHY

GRB2 (1) --------IPKNYIEMKPHPWFFGKIPRA---------KAEEMLSKQRHD-----GAFLIRESESAPG--DFSLSVKFGN---------------DVQHF

GRAP2 (1) ---------PKNYIRVKPHPWYSGRISRQ---------LAEEILMKRNHL-----GAFLIRESESSPG--EFSVSVNYGD---------------QVQHF

NCK1 (1) --------RPSLTGKFAGNPWYYGKVTRH---------QAEMALNERGHE-----GDFLIRDSESSPN--DFSVSLKAQG---------------KNKHF

NCK2 (1) -------TGPSSSGRFAGREWYYGNVTRH---------QAECALNERGVE-----GDFLIRDSESSPS--DFSVSLKASG---------------KNKHF

PTPN6-N (1) ---------------MLSRGWFHRDLSGL---------DAETLLKGRGVH-----GSFLARPSRKNQG--DFSLSVRV------------GD---QVTHI

PTPN11-N (1) ---------------MTSRRWFHPNITGV---------EAENLLLTRGVD-----GSFLARPSKSNPG--DFTLSVRR------------NG---AVTHI

PTPN6-C (1) -------KYPLNCSDPTSERWYHGHMSGG---------QAETLLQAKGEP-----WTFLVRESLSQPG--DFVLSVLSDQPKAGP-----GS-PLRVTHI

PTPN11-C (1) ---------PLNCADPTSERWFHGHLSGK---------EAEKLLTEKGKH-----GSFLVRESQSHPG--DFVLSVRTGDDKGES-----NDGKSKVTHV

SH2D2A (1) ----QKTQAHWLLQHGAAPAWFHGFITRR---------EAERLLEPKPQ------GCYLVRFSESAVT---FVLTYRSRT---------------CCRHF

HSH2D (1) RLDWFVHTQMGQLAQDGVPEWFHGAISR---------EDAENLLESQP---L---GSFLIRVSHSHVG---YTLSYKAQS---------------SCCHF

PIK3R1-N (101) KIFHRDG---KYGFSDPL--------------TFSSVVELINHYRNESLAQYNPKLDVKLLYPVSKYQQD---------------------------------

PIK3R3-N (101) KIYHRDG---KYGFSDPL--------------TFNSVVELINHYHHESLAQYNPKLDVKLMYPVSRYQQD---------------------------------

PIK3R2-N (101) KVFHRDG---HYGFSEPL--------------TFCSVVDLINHYRHESLAQYNAKLDTRLLYPVSKYQQD---------------------------------

PIK3R2-C (101) VIYRTAT---GFGFAEPYN-------------LYGSLKELVLHYQHASLVQHNDALTVTLAHPVRAPGPGPPPAAR---------------------------

PIK3R1-C (101) VINKTAT---GYGFAEPYN-------------LYSSLKELVLHYQHTSLVQHNDSLNVTLAYPVYAQQRR---------------------------------

PIK3R3-C (101) VIYSTAR---GYGFAEPYN-------------LYSSLKELVLHYQQTSLVQHNDSLNVRLAYPVHAQMPSLCR------------------------------

VAV1 (101) KIMTAEG---LYRITEKK--------------AFRGLTELVEFYQQNSLKDCFKSLDTTLQFPFKEPEKR----------------------------------

VAV3 (101) KILTRDG---FFHIAENR--------------KFKSLMELVEYYKHHSLKEGFRTLDTTLQFPYKEPEHSAG--------------------------------

VAV2 (101) KVVEKDN---WIHITEAK--------------KFDSLLELVEYYQCHSLKESFKQLDTTLKYPYKSRERSA---------------------------------

PLCG2-C (101) RINRDGRHFVLGTSAY-----------------FESLVELVSYYEKHS------LYRKMRLRYPVTPELLERYNMERDI------------------------

PLCG1-C (101) RVQQEGQTVMLGNSE------------------FDSLVDLISYYEKHP------LYRKMKLRYPINEEALEKIGT----------------------------

CRK (101) IINSSGPRPPVPPSPAQPPPGVSPSRLRIGDQEFDSLPALLEFYKIHYLDTTTLIEPVSRSRQG-SGVIL----------------------------------

CRKL (101) IINSLPNR-------------R----FKIGDQEFDHLPALLEFYKIHYLDTTTLIEPAPRYPSPPMGSVS----------------------------------

SOCS7 (101) RMEHYRG-TFSLWCHPKFED------------RCQSVVEFIKRAIMHSKNGKFLYFLRSRVP-----------------------------------------

SOCS6 (101) RIEHSNG-RFSFYEQPDVEG-------------HTSIVDLIEHSIRDSENGAFCYS-RSRLPGSATY------------------------------------

SOCS5 (101) RIEQWNH-NFSFDAHDPCVF------------HSSTVTGLLEHYKDPSSCMFFEPLLTISLNRTFPFSL----------------------------------

SOCS4 (101) RIEQWNH-NFSFDAHDPCVF------------HSPDITGLLEHYKDPSACMFFEPLLSTPLIRTFPF------------------------------------

SOCS3 (101) RIQCEGGSFSLQSDPRSTQP----------VPRFDCVLKLVHHYMPPPGAPSFPSPPTEPSSEVPEQPS----------------------------------

SOCS1 (101) RVHFQAGRFHLDGSRE----------------SFDCLFELLEHYVAAPRRMLGAPLRQRRVRPLQELCRQ---------------------------------

SOCS2 (101) RIEYQDG-KFRLDSIICVKSK--------LKQ-FDSVVHLIDYYVQMCKDKRTGPEAPRNGTVHLYLTKP---------------------------------

CISH (101) RIEYADS-SFRLDSNCLSRPR--------ILA-FPDVVSLVQHYVASCTADTRSDSPDPAPTPAL--------------------------------------

FGR (101) KIRKLDMGGYYITTR----------------VQFNSVQELVQHYMEVNDGLCNLLIAPCTIMKPQTLGLAKDA-------------------------------

YES1 (101) KIRKLDNGRYYITTR----------------EQLDTLQKLAKHYTEHADGLCHKLTTVCPTVKPQIQGLA----------------------------------

FYN (101) KIRKLDNGGYYITTR----------------AQFETLQQLVQHYSGTWNGNTKVAIKTLKPGTMSPESFLEEAQ------------------------------

SRC (101) KIRKLDSGGFYITSR----------------TQFNSLQQLVAYYSKHADGLCHRLTTVCPTSKPQTQGL-----------------------------------

LCK (101) KIRNLDNGGFYISPR----------------ITFPGLHELVRHYTNASDGLCTRLSRPCQTQKPQKPWWED---------------------------------

HCK (101) KIRTLDNGGFYISPR----------------STFSTLQELVDHYKKGNDGLCQKLSVPCMSSKPQKPWEKD---------------------------------

LYN (101) KIRSLDNGGYYISPR----------------ITFPCISDMIKHYQKQADGLCRRLEKACISPKPQKPWD-----------------------------------

BLK (101) KIRCLDEGGYYISPR----------------ITFPSLQALVQHYSKKGDGLCQRLTLPCVRPAPQNPWAQ----------------------------------

FRK (101) RIKRLDEGGFFLTRR----------------RIFSTLNEFVSHYTKTSDGLCVKLGKPCLKIQVPAPFDLSY--------------------------------

SLA (101) RIFRLPNNWYYISPR----------------LTFQCLEDLVNHYSEVADGLCCVLTTPCLTQSTAAPAV-----------------------------------

SLA2 (101) RIHCLDNGWLYISPR----------------LTFPSLQALVDHYSELADDICCLLKEPCVLQRAGPLPG-----------------------------------

ABL2 (101) RINTTADGKVYVTAE----------------SRFSTLAELVHHHSTVADGLVTTLHYPAPKCNKPTVYGVSPIHD-----------------------------

ABL1 (101) RINTASDGKLYVSSE----------------SRFNTLAELVHHHSTVADGLITTLHYPAPKRNKPTVYGVSPNY------------------------------

INPPL1 (101) RILPDGEDFLAVQTSQG-----------VPVRRFQTLGELIGLYAQPNQGLVCALLLPVEGEREPDPPDDRDA------------------------------

SH2D1A (101) RVSQTETGSWSAETAPG-----------VHKRYFRKIKNLISAFQKPDQGIVIPLQYPVEKKSSA--------------------------------------

EAT2 (101) RIFREKHGYYRIQTAEG-----------SPKQVFPSLKELISKFEKPNQGMVVHLLKPIKRTSPSLRW------------------------------------

DAPP1 (101) HVEYTGYSFKFGFN------------------EFSSLKDFVKHFANQPLIGSETGTLMVLKHPYPRKVEEPSIYESVRV------------------------

SH2D3C (101) KINKVVVKAG---------ESYTHIQYLFEQESFDHVPALVRYHVGSRKAVSEQSGAIIYCPVNRTFPLR---------------------------------

BCAR3 (101) KINRTVLRLS---------EAYSRVQYQFEMESFDSIPGLVRCYVGNRRPISQQSGAIIFQPINRTVPLR---------------------------------

SH2D3A (101) EVFRVALRPRPGRP---------TALFQLEDEQFPSIPALVHSYMTGRRPLSQATGAVVSRPVTWQGPL----------------------------------

FER (101) IIQYVDN------------------MYRFEGTGFSNIPQLIDHHYTTKQVITKKSGVVLLNPIPKDKKWILS--------------------------------

FES (101) IIQSLDN------------------LYRLEGEGFPSIPLLIDHLLSTQQPLTKKSGVVLHRAVPKDK-------------------------------------

E105251 (101) KFARTRENQVVLGQHSGP---------------FPSVPELVLHYSSRPLPVQGAEHLALLYPVVTQTP-----------------------------------

SHB (101) KLAKTKE-KYVLGQNSPP---------------FDSVPEVIHYYTTRKLPIKGAEHLSLLYPVAVRTL------------------------------------

E138606 (101) KLSRTKEHKYVLGQNSPP---------------FSSVPEIVHHYASRKLPIKGAEHMSLLYPVAIRTL-----------------------------------

E169291 (101) IVAQTKDNKYTLNQTSAV---------------FDSIPEVVHYYSNEKLPFKGAEHMTLLYPVHSKLH-----------------------------------

SH2B (101) RLSLNEEG-----------------QCRVQHLWFQSIFDMLEHFRVHPIPLESGGSSDVVLVSYVPSSQRQQGEQ-----------------------------

APS (101) RLSLNGHG-----------------QCHVQHLWFQSVLDMLRHFHTHPIPLESGGSADITLRSYVRAQDPPPE-------------------------------

LNK (101) RLSLTERG-----------------QCRVQHLHFPSVVDMLHHFQRSPIPLECGAACDVRLSSYVVVVSQPPGSCNTVLFPFSLPHWDSESLPHWGSELG----

SHC1 (101) LLVDPEG------------------VVRTKDHRFESVSHLISYHMDNHLPIISAGSELCLQQPVERKL------------------------------------

E185634 (101) LLVDPEG-----K-------------VRTKDHVFDNVGHLIRYHMDNSLPIISSGSE----------------------------------------------

SHC3 (101) LLVDPEG------------------TIRTKDRVFDSISHLINHHLESSLPIVSAGSELCLQQPVERKQ------------------------------------

RASA1-N (101) RIIAMCGDYYIGGRR------------------FSSLSDLIGYYSHVSCLLKGEKLLYPVAPPEAIEDRRR--------------------------------

PLCG1-N (101) RIHSRQDAGT------------PKFFLTDNLVFDSLYDLITHYQQVPLRCNEFEMRLSEPVPQTNAHE-----------------------------------

PLCG2-N (101) RIRSTMEGGT------------LKYYLTDNLTFSSIYALIQHYRETHLRCAEFELRLTDPVPNPNPHESKP--------------------------------

RASA1-C (101) KICPTPNNQFMMGGR-----------------YYNSIGDIIDHYRKEQIVEGYYLKEPVPMQDQEQVLND---------------------------------

RIN3 (101) LEYTIKEEKSILYLEGSA-------------LVFEDIFRLIAFYCVSRDLLPFTLRLPQAILEASSFT------------------------------------

RIN2 (101) KEFAIKESTYTFSLEGSG-------------ISFADLFRLIAFYCISRDVLPFTLKLPYAISTAKS--------------------------------------

RIN1 (101) ILESPGGVSLEGS-E----------------LMFPDLVQLICAYCHTRDILLLPLQLPRAIHHAATHKELE---------------------------------

TENC1 (101) RHFLIETGPKGVKIKGCPSE-----------PYFGSLSALVSQHSISPISLPCCLRIPSKDPLEETPEAPVPTNM----------------------------

TENS1 (101) RHFLIECTPKGVRLKGCSNE-----------PYFGSLTALVCQHSITPLALPCKLLIPERDPLEEIAES----------------------------------

TNS (101) RHFLIETGPRGVKLKGCPNE-----------PNFGSLSALVYQHSIIPLALPCKLVIPNRDPTDESKDSS----------------------------------

CTEN (101) RHFLIESSAKGVHLKGADEE-----------PYFGSLSAFVCQHSIMALALPCKLTIPQRELGGADG-------------------------------------

JAK1 (101) QIEVQK-GRYSLHGSDRS---------------FPSLGDLMSHLKKQILRTDNISFMLKRCCQPKPREISNLLVATKKAQEWQ---------------------

JAK2 (101) LITKNENEEYNLSGTKKN---------------FSSLKDLLNCYQMETVRSDNIIFQFTKCCPPKPKDKSNL--------------------------------

JAK3 (101) LIRRSPTGTFLLVGLSRP---------------HSSLRELLATCWDGGLHVDGVAVTLTSCCIPRPKEKSNLI-------------------------------

BRDG1 (101) KVMSVGQNYTIELEKP---------------VTLPNLFSVIDYFVKETRGNLRPFICSTDENTGQEPSMEGRS------------------------------

E109111 (101) DVREEGKENAFSLGATLWIN----------SEEFEDLDEIVARYVQPMASFARDLLNHKYYQDCSGGDRKKLEELLIKTKKEKPTFIPY--------------

STAT1 (101) VEPYTKKELSAVTFPDIIRNY--------KVMAAENIPENPLKYLYPNIDKDHAFGKYYSRPKEAPEPMELDGPKG---------------------------

STAT4 (101) VEPYNKGRLSALPFADILRDY--------KVIMAENIPENPLKYLYPDIPKDKAFGKHYSS-----QPCE---------------------------------

STAT3 (101) VEPYTKQQLNNMSFAEIIMGY--------KIMDATNILVSPLVYLYPDIPKEEAFGKYCRP-----ESQ----------------------------------

STAT2 (101) VQPYTKEVLQSLPLTEIIRHY--------QLLTEENIPENPLRFLYPRIPRDEAFGCYYQEKVFR--------------------------------------

STAT5a (101) LKPFTTRDFSIRSLADRLG---------------D---LSYLIYVFPDRPKDEVFSKYYTPVLAK--------------------------------------

STAT6 (101) IQPFSAKDLSIRSLGDRIR---------------D---LAQLKNLYPKKPKDEAFRSHYKPE-----------------------------------------

CHN1 (101) RLYYDGKHFVG------------------E-KRFESIHDLVTDGLITLYIETKAAEYIAKMTINPIYEHVGYTTLNREPAYKKHMPVLKETH------------

CHN2 (101) RLFHDGKHFVG------------------E-KRFESIHDLVTDGLITLYIETKAAEYISKMTTNPIYEHIGYATL-----------------------------

PTK6 (101) KIWRRAGGRLHLN----------------EAVSFLSLPELVNYHRAQSLSHGLRLAAPCRKHEPEPLPHWD---------------------------------

E18941 (101) QVFRNHLGRYCLEHLP---------------AEFPSLEALVENHAVTERSLFCPLDMGRLNPTYEEQD-----------------------------------

TEC (101) HIKETTTSP---K-----------KYYLAEKHAFGSIPEIIEYHKHNAAGLVTRLRYPVSVKGKNAPTTAG---------------------------------

BTK (101) VVCSTPQS----------------QYYLAEKHLFSTIPELINYHQHNSAGLISRLKYPVSQQNKNAPSTAGLGYGSWE--------------------------

ITK (101) HIKETNDNP---K-----------RYYVAEKYVFDSIPLLINYHQHNGGGLVTRLRYPVCFGRQKAPVTAG---------------------------------

TXK (101) QIKKNDSG----------------QWYVAERHAFQSIPELIWYHQHNAAGLMTRLRYPVGLMGSCLPAT-----------------------------------

BMX (101) HVHTNAEN----------------KLYLAENYCFDSIPKLIHYHQHNSAGMITRLRHPVSTKANKVPDSVS---------------------------------

LCP2 (101) QIRYQKESQVYLLGTGLR-----------GKEDFLSVSDIIDYFRKMPLLLIDGKNRGSRYQCTLTHAAGYP--------------------------------

MIST (101) KIRFLERNQQFALGTGLRG-----------DEKFDSVEDIIEHYKN----------------------------------------------------------

BLNK (101) PVRFIEATKQYALGRKKN-----------GEEYFGSVAEIIRNHQHSPLVLIDSQNNTKDSTRLKYAVKVS---------------------------------

SH3BP2 (101) RIFEKDSKFYLEG-----------------EVLFVSVGSMEHYHTHVLPSHQSLLLRHPYGYTGPR-------------------------------------

SYK-N (101) TIERELNGTYAIAGG----------------RTHASPADLCHYHSQESDGLVCLLKKPFNRPQGVQ-------------------------------------

ZAP70-N (101) PIERQLNGTYAIAGG----------------KAHCGPAELCEFYSRDPDGLPCNLRKPCNRPSGLEPQPGVFDCLRD--------------------------

GRB14 (101) QIIPVEDDGE------------MFHTLDDGHTRFTDLIQLVEFYQLNKGVLPCKLKHYCARIAL---------------------------------------

GRB10 (101) QILPCEDDGQ------------TFFSLDDGNTKFSDLIQLVDFYQLNKGVLPCKLKHHCIRVAL---------------------------------------

GRB7 (101) LILPSEEEGR------------LYFSMDDGQTRFTDLLQLVEFHQLNRGILPCLLRHCCTRVAL----------------------------------------

ZAP70-C (101) LISQDKAGKYCIPEG----------------TKFDTLWQLVEYLKLKADGLIYCLKEACPN-SSASNASGAAAP-----------------------------

SYK-C (101) RIDKDKTGKLSIPEG----------------KKFDTLWQLVEHYSYKADGLLRVLTVPCQK-IGTQGNVNFGGR-----------------------------

MATK (101) RVLHRDGH-----------------LTIDEAVFFCNLMDMVEHYSKDKGAICTKLVRPKRKHGTKSAEEELA--------------------------------

CSK (101) RIMYHASK-----------------LSIDEEVYFENLMQLVEHYTSDADGLCTRLIKPKVMEGTVAAQ------------------------------------

GRB2 (101) KVLRDGAGKYFLWVVK-----------------FNSLNELVDYHRSTSVSRNQ-QIFLRDIEQVPQQPTY----------------------------------

GRAP2 (101) KVLREASGKYFLWEEK-----------------FNSLNELVDFYRTTTIAKKR-QIFLRDEEPLLKSPGA---------------------------------

NCK1 (101) KVQLKET-VYCIGQRK-----------------FSTMEELVEHYKKAPIFTSE-QGEKLYLVKHLS--------------------------------------

NCK2 (101) KVQLVDN-VYCIGQRR-----------------FHTMDELVEHYKKAPIFTSE-HGEKLYLVRALQ--------------------------------------

PTPN6-N (101) RIQNSGDFYDLYGGEK-----------------FATLTELVEYYTQQQGVLQDRDGTIIHLKYPLNC------------------------------------

PTPN11-N (101) KIQNTGDYYDLYGGEK-----------------FATLAELVQYYMEHHGQLKEKNGDVIELKYPLN-------------------------------------

PTPN6-C (101) KVMCEGGRYTVGGLET-----------------FDSLTDLVEHFKKTG--IEEASGAFVYLRQPYYATRVNAA------------------------------

PTPN11-C (101) MIRCQELKYDVGGGER-----------------FDSLTDLVEHYKKNP--MVETLGTVLQLKQPLNTTRIN--------------------------------

SH2D2A (101) LLAQLRDGRHVVLGEDS---------------AHARLQDLLLHYTAHPLSPYGETLTEPLARQTPEPAGLSLRTEESNFGSKSQDPNPQYSPIIKQGQAPVPMQKE

HSH2D (101) MVKLLDDGTFMIPGEK---------------VAHTSLDALVTFHQQKPIEPRRELLTQPCRQKDPAN------------------------------------

Consensus (101) RI F SL ELV HY L
